# MsmR1, a global transcription factor, regulates polymyxin synthesis and carbohydrate metabolism in *Paenibacillus polymyxa* SC2

**DOI:** 10.3389/fmicb.2022.1039806

**Published:** 2022-11-22

**Authors:** Dongying Zhao, Hui Li, Yanru Cui, Shengyue Tang, Chengqiang Wang, Binghai Du, Yanqin Ding

**Affiliations:** College of Life Sciences and Shandong Engineering Research Center of Plant-Microbia Restoration for Saline-alkali Land and Shandong Key Laboratory of Agricultural Microbiology and National Engineering Laboratory for Efficient Utilization of Soil and Fertilizer Resources, Shandong Agricultural University, Tai’an, China

**Keywords:** *Paenibacillus polymyxa*, MsmR1, polymyxin, carbohydrate metabolism, ChIP-seq

## Abstract

The multiple-sugar metabolism regulator (MsmR), a transcription factor belonging to the AraC/XylS family, participates in polysaccharide metabolism and virulence. However, the transcriptional regulatory mechanisms of MsmR1 in *Paenibacillus polymyxa* remain unclear. In this study, knocking out *msmR1* was found to reduce polymyxin synthesis by the SC2-M1 strain. Chromatin immunoprecipitation assay with sequencing (ChIP-seq) revealed that most enriched pathway was that of carbohydrate metabolism. Additionally, electromobility shift assays (EMSA) confirmed the direct interaction between MsmR1 and the promoter regions of *oppC3*, *sucA*, *sdr3*, *pepF*, *yycN*, *PPSC2_23180*, *pppL*, and *ydfp*. MsmR1 stimulates polymyxin biosynthesis by directly binding to the promoter regions of *oppC3* and *sdr3*, while also directly regulating *sucA* and influencing the citrate cycle (TCA cycle). In addition, MsmR1 directly activates *pepF* and was beneficial for spore and biofilm formation. These results indicated that MsmR1 could regulate carbohydrate and amino acid metabolism, and indirectly affect biological processes such as polymyxin synthesis, biofilm formation, and motility. Moreover, MsmR1 could be autoregulated. Hence, this study expand the current knowledge of MsmR1 and will be beneficial for the application of *P. polymyxa* SC2 in the biological control against the certain pathogens in pepper.

## Introduction

*Paenibacillus polymyxa*, proposed by Ash et al. (1993), is a plant growth-promoting rhizobacteria (PGPR) that contributes to disease prevention and growth promotion by inducing plant systemic resistance, resulting in generation of secondary metabolites (Li et al., 2019; Liu et al., 2021; Yin et al., 2022). *Paenibacillus polymyxa* is an important strain resource in microbial medicine, microbial environmental remediation, microbial pesticides, and fertilizer research (Timmusk et al., 2005; Jeong et al., 2019). *Paenibacillus polymyxa* synthesizes polymyxin—an important antibiotic against gram-negative pathogens—*via* a multienzyme non-ribosomal peptide synthetase system (NRPS; Supplementary Figure S1A; Yu et al., 2015, 2018). Polymyxin is composed of 10 orderly assembled amino acid residues, five of which are L-2,4-diaminobutyric acid (L-Dabs) biosynthesized by 2,4-diaminobutyrate aminotransferase (EctB) (Choi et al., 2009). Moreover, polymyxins are primarily divided into seven types: A, B, C, D, E, M, and P, composed of a tripeptide side chain acylated by a fatty acid at the amino-terminus, and a cyclic heptapeptide (Supplementary Figure S1B). Indeed, carbon sources significantly impact the rate of polymyxin production and biosynthesis, for example, starch can promote polymyxin synthesis (Yu et al., 2018).

Carbohydrates exist in various forms, including monosaccharides, disaccharides, and polysaccharides. Energy released from carbohydrate catabolism is required for metabolic activity and growth (Warda et al., 2016). Pathways of carbohydrate catabolism involve complex networks that regulate gene expression. In bacteria, carbohydrate utilization is regulated by transcription factors (TFs) regulating the expression of genes which encodes transporters and enzymes (Khoroshkin et al., 2016). Efficient utilization of carbohydrates by bacteria is determined by various utilization systems and carbohydrate transporters (Warda et al., 2016).

Indeed, TFs have essential roles in regulating gene expression in all domains of life (Mejía-Almonte et al., 2020; Cortés-Avalos et al., 2021). More specifically, TFs can activate or repress gene transcription by binding to DNA in a manner dependent on environmental factors (Cortés-Avalos et al., 2021). In bacteria, TFs can be classified into at least 19 families based on their amino acid sequences. The largest TFs families are LysR, TetR/ArcR, and AraC/XylS, which together account for 30% of the total number of TFs identified in bacteria (Kotecka et al., 2021). The AraC/XylS family of TFs was originally described by Henikoff et al. (1990); members of this family act as positive transcriptional regulators or as both activators and repressors. Previous studies focusing on the TFs of RhaR (Kolin et al., 2008; Kettle, 2013), AraC (Schleif, 2010), UreR (Parra and Collins, 2012), and MelR (Belyaeva et al., 2000) in *Escherichia coli*, MsmR (Nakata et al., 2005) in *Streptococcus*, XylS (Zwick et al., 2013) in *Pseudomonas putida*, and Toxt (Lowden et al., 2010) in *Vibrio cholerae* revealed that they contain a highly conserved C-terminal DNA binding domain (DBD) and a variable ligand-binding domain (LBD) at the N-terminal (Bustos and Schleif, 1993; Cortés-Avalos et al., 2021). The DBD contains approximately 100 amino acids, with approximately 20% sequence identity in bacteria, and seven α-helices, forming two helix-turn-helix (HTH) DNA-binding motifs (Egan, 2002; Gahlot et al., 2021). While the DBD is in contact with RNA polymerase (RNAP) for DNA binding and transcription (Ibarra et al., 2008), the LBD is involved in dimerization and effector/signal recognition (Egan, 2002). The AraC/XylS family transcriptional regulators control multiple functions, such as carbon metabolism, amino acid metabolism, stress response (e. g. Rob, SoxS, PliA, and OpiA were involved in stress response in *Erwinia amylovora*; Pletzer et al., 2014), and quorum sensing (Cortés-Avalos et al., 2021; Kotecka et al., 2021).

The MsmR of the AraC/XylS family is involved in polysaccharide metabolism (Russell et al., 1992). In *Streptococcus* mutants, MsmR positively regulates an operon for the uptake and catabolism of carbon sources (Russell et al., 1992). In *Streptococcus pyogenes*, MsmR positively regulates fibronectin-and collagen-binding T-antigen (FCT) region genes, including *nra*, which inhibit the expression of crucial virulence factors (Kreikemeyer et al., 2007). Meanwhile, the transcriptional regulatory mechanisms of MsmR1 in *P. polymyxa* remain unclear.

*Paenibacillus polymyxa* SC2 was isolated from the rhizosphere of pepper plants in Guizhou province, China (Ma et al., 2011). *Paenibacillus polymyxa* SC2-M1 is spontaneously mutated from *P*. *polymyxa* SC2 during strain cultivation, making it suitable for molecular manipulations (Hou et al., 2016). A previous study reported that the *msmR1* gene is upregulated by 2.17-fold during the interaction between *P. polymyxa* SC2 and pepper (Liu et al., 2021), indicating that *msmR1* may play an important role in this interaction. In the present study, we aimed to explore the regulatory mechanism of the transcription factor MsmR1 in polymyxin synthesis and related biological processes in *P. polymyxa* SC2. To this end, chromatin immunoprecipitation with sequencing (ChIP-seq) was performed and the results were verified by electrophoretic mobility shift assay (EMSA). The *oppC3*, *sucA*, *sdr3*, *pepF*, *yycN*, *PPSC2_23180*, *pppL*, and *ydfp* genes were found to be directly regulated by the global regulator MsmR1; the functions of these genes were further explored to delineate the regulatory mechanism of MsmR1. The results of this study provide a theoretical basis for the improved application of *P. polymyxa* SC2 in the biological control against the certain pathogens.

## Materials and methods

### Bacterial strains and culture conditions

The strains and plasmids used in this work are presented in Supplementary Table S1. *Paenibacillus polymyxa* SC2-M1 and its mutants, *Escherichia coli* DH5α, *E. coli* BL21, and Trans 110 were cultured in Luria-Bertani (LB) broth at 37°C. When plasmids were presented in cells, antibiotics were added to LB medium as follows: chloramphenicol (6 μg/ml for strain SC2-M1 and its mutants), kanamycin (50 μg/ml), tetracycline (20 μg/ml), ampicillin (100 μg/ml), and erythromycin (12.5 μg/ml). To measure carbon source utilization by the strains, basal medium (MgSO_4_ 0.02%, NaCl 0.02%, (NH_4_)_2_SO_4_ 0.8%, K_2_HPO_4_ 0.368%, KH_2_PO_4_ 0.132%) with a 1% sole carbon source was used.

Competent cells of *E. coli* were prepared using the transformation and storage solution (TSS) method and transformed by the heat pulse method (Chung et al., 1989). Competent cells were prepared and *P. polymyxa* SC2-M1 were electrotransformed as previously described (Hou et al., 2016).

### Plasmid and strain construction

To construct knockout SC2-M1 strain mutants, 650–1,000 bp of homologous arm-flanking target genes were cloned from genomic DNA using PCR. The *cat* gene, encoding the chloramphenicol resistance cassette, was cloned from pDG1661 (Guérout-Fleury et al., 1996) using PCR to replace the target knockout genes. Two homologous arms and *cat* cassettes were fused by fusion PCR (Cha-Aim et al., 2012), and the fusion DNA fragments were ligated into the thermo-sensitive elimination plasmid pRN5101 to construct knockout vectors. The plasmid was demethylated by being further transformed into Trans 110. The demethylated knockout vectors were transferred into the SC2-M1 strain. Double-crossover recombinants were detected as previously reported (Hou et al., 2016), and the knockout strains were confirmed by PCR using the corresponding primers (Supplementary Table S2). For *in situ* complementation of the knockout genes, 650–1,000 bp of homologous arms fused with the corresponding complete target genes were cloned by PCR from genomic DNA (not involved in the resistance cassette), and ligated into pRN5101 to construct the complement vectors. Demethylated complementary vectors were introduced into the mutant strains. Double-crossover recombinants were detected as described above, and the complemented strains were confirmed by PCR using the corresponding primers (Supplementary Table S2). For ChIP-seq analysis, we obtained a Flag-tagged strain using homologous recombination. The Flag-tag was added in the front of termination codon of *msmR1* and a chloramphenicol resistance cassette was also added after the Flag tag using overlapping PCR. The homologous arms (650–1,000 bp) were cloned by PCR amplification, and ligated to pRN5101 to construct the plasmid pRN5101-*msmR1*-Flag. Demethylated pRN5101-*msmR1*-Flag were introduced into SC2-M1 and double-crossover recombinants were detected as described above, designated as strain SC2-M1-Flag. For overexpression of target genes, fragments carrying complete target genes were cloned from genomic DNA, fused with the promoter fragments, and ligated into pHY300PLK between *Bgl*II and *Sal*I sites by ClonExpress MultiS One Step Cloning Kit (Vazyme Biotech, Nanjing, China) for overexpression in SC2-M1. To evaluate the promoter activity of target DNA fragments, the fragments were cloned from genomic DNA, and the promoterless green fluorescent protein (gfp) fragment from pGFP4412 was cloned (Li et al., 2019). The two fragments were fused using PCR and ligated into pHY300PLK between *Bgl*II and *Sal*I sites as described above, thus generating the pHY300PLK-promoter-gfp plasmid.

### Chromatin immunoprecipitation assay with sequencing (ChIP-seq)

The SC2-M1 and SC2-M1-Flag strains were cultured in 50 ml of LB medium at 37°C with shaking at 180 rpm for 10 h, inoculated with an initial OD_600_ of 0.8 in 50 ml of LB broth by 2% (v/v), and cultured at 37°C with shaking at 180 rpm for 7 h. The two strains were then fixed with 1% formaldehyde for 10 min at 28°C and the reaction was stopped with 125 mM glycine for 5 min. The cells of the two strains were washed thrice with phosphate buffer solution (PBS) at 8000 rpm for 5 min at 4°C and stored at-80°C. ChIP was performed by Wuhan IGENEBOOK Biotechnology Co. Ltd., China. The manufacturer’s instructions of Illumina TruSeq ChIP Sample Prep Set A were followed to construct sequencing libraries using chromatin immunoprecipitated DNA which was then sequenced on an Illumina Xten using the PE 150 method (Chen et al., 2021). The obtained reads were filtered by Trimmomatic (v 0.30). Clean reads were then mapped to the *P. polymyxa* SC2 genome using Bwa (v 0.7.15), which allowed for up to two mismatches (Pjanic, 2017). MsmR1 peaks were produced and visualized using MACS software and Integrative Genomics Viewer (IGV, v 2.3.91), respectively (Zhang et al., 2008; Thorvaldsdóttir et al., 2013). To understand the potential roles of genes, Kyoto Encyclopedia of Genes and Genomes (KEGG) and multiple EM for motif elicitation (MEME) were used for pathway enrichment analysis and motif analysis, respectively (Chen et al., 2021; Wang et al., 2022).

### Quantitative real-time PCR

SteadyPure Universal RNA Extraction Kit (Accurate Biology, Hunan, China) was used to purify the total RNA from the target bacteria. cDNA was obtained from 1 μg of total RNA using the Evo M-MLV RT Kit with gDNA clean for qPCR II (Accurate Biology, Hunan, China). qRT-PCR analysis was performed on a Roter-Gene Q PCR system using the SYBR Green Pro Taq HS qPCR Kit (Accurate Biology, Hunan, China). The relative expression of target genes was calculated using the 2^−ΔΔCt^ method (Liu et al., 2021). The housekeeping gene glyceraldehyde-3-phosphate dehydrogenase (*gapdh*) was used as an internal control.

### Purification of MsmR1 protein and electromobility shift assays

An 828 bp fragment (275 amino acids) of *msmR1* was cloned from the genomic DNA of strain SC2-M1 using primers MsmR1F and MsmR1R (Supplementary Table S2). The fragments of *msmR1* and plasmid pET-28b(+) were simultaneously cleaved with *Xho*I and *Sal*I, purified, ligated using T4 DNA ligase, and transferred into *E. coli* DH5α to obtain pET28b(+)-*msmR1*.

To overexpress the MsmR1 protein, the plasmid pET28b(+)-*msmR1* was transferred into *E. coli* BL21. The transformants were cultured in kanamycin-resistant LB broth at 37°C with shaking at 180 rpm for 10 h. Next, the cells were inoculated into 50 ml of kanamycin-resistant LB broth at 2% (v/v) and cultured at 37°C with shaking at 180 rpm for approximately 1.2 h until the OD_600_ reached 0.5–0.6. Then the cells were induced by 0.4 mM IPTG at 16°C for 20 h, harvested, washed, resuspended in PBS, and sonicated on ice. MsmR1-His6 protein was purified using BeyoGold™ His-tag Purification Resin (Beyotime, China) following the manufacturer’s instructions. The purified protein was added to a final concentration of 10% (v/v) glycerol and stored at-80°C. EMSA was performed using a chemiluminescent EMSA kit (Beyotime, China; Zhang et al., 2021). The concentration of labeled probes and competitive probes were about 0.4 and 4 μM, respectively. The gradient concentration of protein were set up for the EMSA experiment.

### Detection of the fluorescence intensity of promotors

Promoter reporter plasmids were transformed into *E. coli* DH5α cells. The strains were inoculated in LB plate containing 20 μg/ml tetracycline and fluorescence was observed with an OLYMPUS fluorescence microscope at 1000× (Li et al., 2019). The fluorescence intensity of the promoters was detected at different time points as previously reported (Li et al., 2019).

### Antibacterial activity analysis

An Oxford cup assay was used to evaluate polymyxin production by the SC2-M1 strain, as previously described (Liu et al., 2021). Bacteria were inoculated in liquid LB medium and cultured at 37°C with shaking at 180 rpm for 10 h. Bacteria were then inoculated with an initial OD_600_ of 0.8 in 50 ml fermentation medium (sucrose 40 g/l, (NH_4_)_2_SO_4_ 8 g/l, CaCO_3_ 5 g/l, KH_2_PO_4_ 0.2 g/l, MgSO_4_ 0.2 g/l, NaCl 0.2 g/l) at 2% (v/v) and cultured at 37°C with shaking at 180 rpm for 48 h.

### Motility assay

A motility assay was performed as previously reported (Guo et al., 2019). Bacteria were cultured on LB medium for approximately 12 h, after which the OD_600_ was adjusted to 0.6. Diluted cell solutions (2 μl) were added to the center of the plate containing 0.5% glucose, 0.22% NaCl, 0.13% yeast extract, 0.45% tryptone, and 0.5% agar.

### Growth curve assay

Bacteria were cultured overnight in liquid LB medium and adjusted to an OD_600_ of 0.8. Diluted strain solutions were inoculated at 2% in 50 ml of fresh LB medium. The bacteria were cultured at 37°C with shaking at 180 rpm. The OD_600_ and number of live bacterial cells were measured every 3 h. A total of 100 μl of the strain solution was serially diluted with 900 μl of sterile water; 100 μl of the dilution was then spread on an LB plate. The plates were subsequently incubated for 24 h at 37°C.

The growth curves of bacteria on the carbon sources were determined using a basal medium. Bacteria were cultured overnight in LB medium. The cultures were centrifuged, washed thrice with basal medium, and resuspended to inoculate 50 ml of fresh basal medium with 1% sole carbon source; the original OD_600_ value was approximately 0.1. Cultures were incubated at 37°C with shaking at 180 rpm, and growth curves were determined according to OD_600_ every 3 h.

### Statistical analysis

For data analysis, the analysis of variance (ANOVA) and Least Significant Difference (LSD) test (*p* ≤ 0.05) were performed using statistical software SPSS (v 19.0, SPSS Inc., Chicago, IL, USA; Wang et al., 2020).

## Results

### Overview of the transcription factor MsmR1 from *Paenibacillus polymyxa* SC2

**Figure 1:**
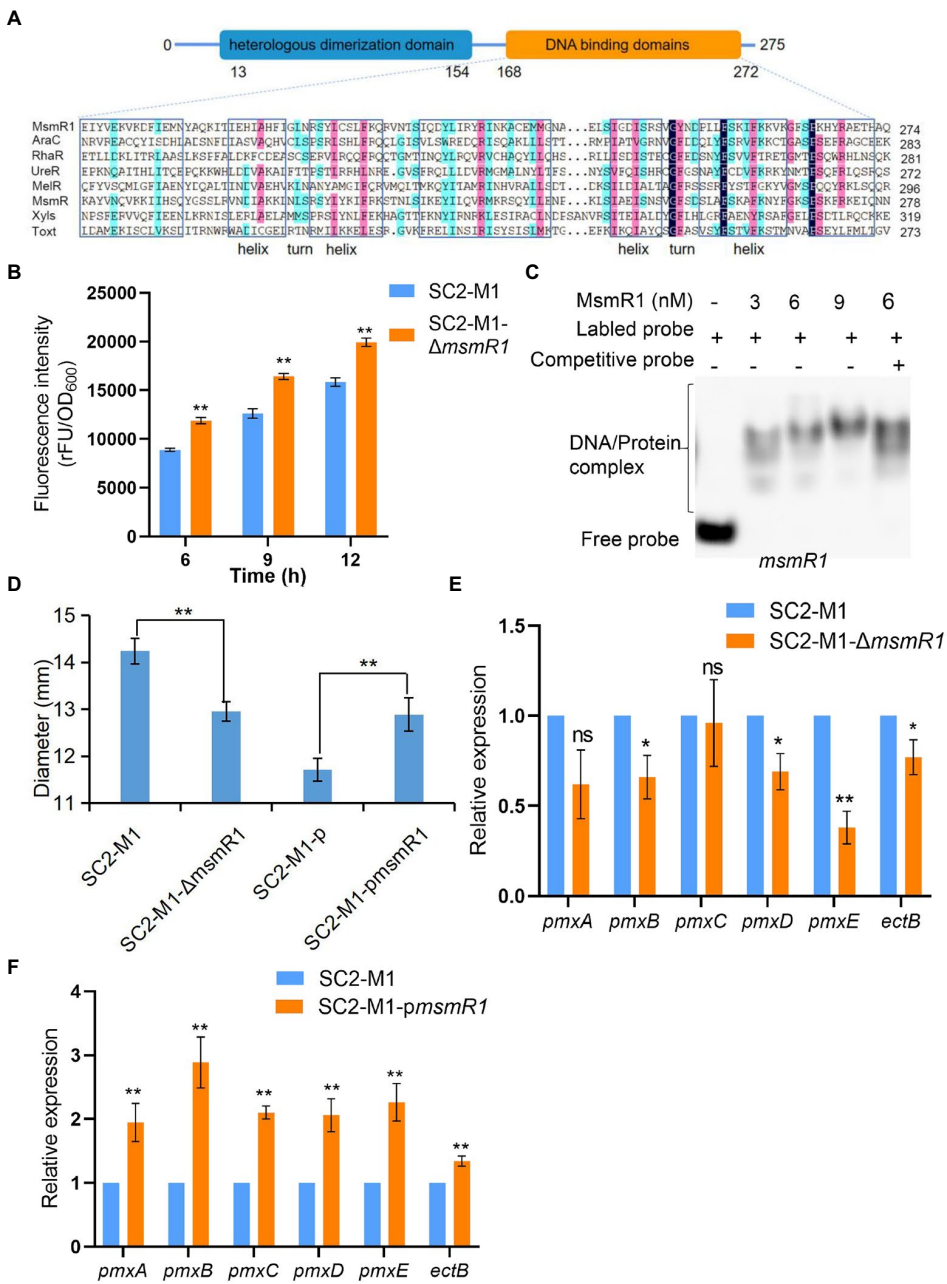
Characteristics of MsmR1 protein in *Paenibacillus polymyxa* SC2-M1. **(A)** Homologous alignment of MsmR1 protein with several AraC family members: AraC (UniProtKB/Swiss-Prot: P0A9E0), RhaR (UniProtKB/Swiss-Prot: P09378), UreR (UniProtKB/Swiss-Prot: P32326), MelR (UniProtKB/Swiss-Prot: P0ACH8), MsmR (UniProtKB/Swiss-Prot: Q00753), XylS (UniProtKB/Swiss-Prot: P07859), and Toxt (UniProtKB/Swiss-Prot: A5F384). Sequences were aligned by DNAMAN software. **(B)** Fluorescence intensity of *msmR1* promoter in strains SC2-M1 and SC2-M1-Δ*msmR1* detected at different time points. **(C)** EMSA results depicting MsmR1 binding to its own promoter. **(D)** Size of the inhibition zone between the SC2-M1, SC2-M1-Δ*msmR1*, SC2-M1-p, and SC2-M1-p*msmR1* strains was determined using the Oxford cup method. **(E)** Relative expression of genes related to polymyxin synthesis gene cluster in strain SC2-M1 and SC2-M1-Δ*msmR1*. **(F)** Relative expression of genes related to polymyxin synthesis gene cluster in the control strain SC2-M1-p and the overexpression strain SC2-M1-p*msmR1*. *p < 0.05 and **p < 0.01; ns, *p* > 0.05.

The MsmR1 of strain SC2, which is classified as an AraC/XylS family transcriptional regulator, contains two domains: an N-terminal heterologous dimerization domain and a C-terminal DNA-binding domain (Figure 1A). The C-terminus of MsmR1 shows high similarities to that of AraC family members: RhaR, AraC, UreR, MelR, MsmR, XylS, and Toxt. Seven α-helix were identified in the C-terminal domain of MsmR1, which combined to form two helix-turn-helix (HTH) motifs (Figure 1A).

AraC family TFs are often auto-regulated (Sun et al., 2016). Hence, the promoter activity of *msmR1* was evaluated using the pHY300PLK-*msmR1*-gfp plasmid. This plasmid was found to possess promoter activity in *E. coli* DH5α cells. In contrast, the control (pHY300PLK-gfp) showed no fluorescence (Supplementary Figure S2A). The pHY300PLK-*msmR1*-gfp plasmid was introduced into the strains SC2-M1 and SC2-M1-Δ*msmR1* which was a *msmR1* knock out strain obtained *via* homologous recombination (Supplementary Figure S2B). The fluorescence intensity of the strain SC2-M1-Δ*msmR1* was significantly higher than that of the SC2-M1 strain (Figure 1B), revealing that MsmR1 could auto-regulate its transcription. EMSA was further performed to determine whether MsmR1 is capable of direct auto-regulation. MsmR1 was found to specifically bind to its own promoter resulting in a retarded band (Figure 1C), indicating that MsmR1 auto-regulates its expression.

To investigate the function of MsmR1, *msmR1* knock out strain SC2-M1-Δ*msmR1* and overexpression strain SC2-M1-p*msmR1* formed by ligation into pHY300PLK plasmid (Supplementary Figure S2C), were used. Meanwhile, SC2-M1-p was set as the control. Notably, the amount of polymyxin in the mutant SC2-M1-Δ*msmR1* was significantly less than that in the SC2-M1 strain; the diameter of the inhibitory zone was decreased by 9.0% (Figure 1D; Supplementary Figure S2D). The qRT-PCR results revealed that the polymyxin synthetase genes were downregulated in the knockout strain compared with SC2-M1 (Figure 1E). In SC2-M1-p*msmR1*, the inhibitory zone diameter was increased by 10.03% compared with SC2-M1-p (Figure 1D; Supplementary Figure S2D). The results were consistent with the qRT-PCR results which presented a significant increase in expression of polymyxin synthetase genes in SC2-M1-p*msmR1* when compared with that in SC2-M1-p (Figure 1F).

Given that AraC family members are involved in carbohydrate metabolism (Cortés-Avalos et al., 2021), the growth curves of the SC2-M1 and SC2-M1-Δ*msmR1* strains were determined using LB and basal media with sole carbon sources. Although phenotypic differences were noted between the two strains using glucose, xylose, galactose, fructose, and lactose, the differences were not significant (Supplementary Figure S2E).

### Identification of MsmR1 binding genes

To determine the target genes directly regulated by MsmR1 in *P. polymyxa* SC2, ChIP-seq experiments were performed using C-terminal FLAG-tagged *msmR1* in SC2-M1. The qRT-PCR results demonstrated that the expression of *msmR1* was high during the early stage and low during the decline phase (Supplementary Figure S3A). Based on the qRT-PCR results and the growth curve, a culture time of 7 h (mid-logarithmic phase) was selected for subsequent ChIP-seq analysis (Supplementary Figure S3).

**Figure 2:**
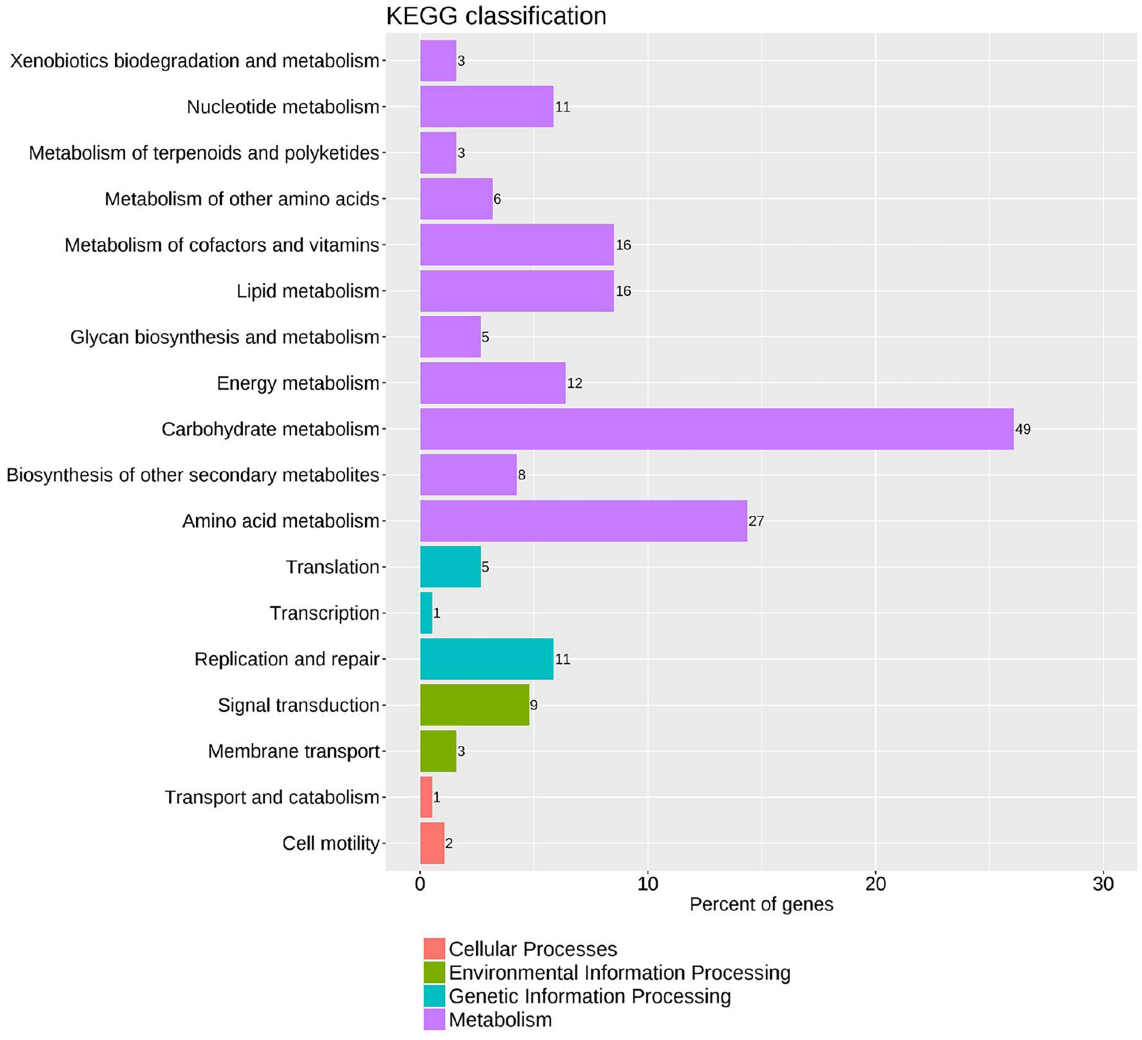
KEGG enrichment analysis of MsmR1 binding DNA according to ChIP-seq data. The fragments obtained from ChIP-seq were analyzed by KEGG, and were enriched in cellular process, environmental information process, genetic information process, and metabolism.

Approximately 3.83–4.86 million reads in the ChIP-seq results were derived from the six samples, which were mapped to the *P. polymyxa* SC2 genome (Supplementary Table S3). Overall, 649 peaks were identified between the two strains, SC2-M1 and SC2-M1-Flag. Among these, 96.15% were located in the coding sequence (CDS) and 3.85% were located in the intergenic regions. To categorize the functions of MsmR1 target genes, KEGG enrichment analysis was performed. The target genes were enriched in various functions, including cellular processes (1.56%), environmental information processing (6.25%), genetic information processing (10.94%), and metabolism (81.25%; Supplementary Table S4). In the metabolism category, carbohydrate metabolism was the most enriched pathway, while amino acid metabolism, metabolism of cofactors and vitamins, lipid metabolism, energy metabolism, and nucleotide metabolism were also significantly enriched (Figure 2). Hence, MsmR1 primarily regulates basal metabolism in SC2 cells. According to the predicted results of MEME, we selected the fragments related to primary metabolism for further verification (Supplementary Table S5).

**Figure 3:**
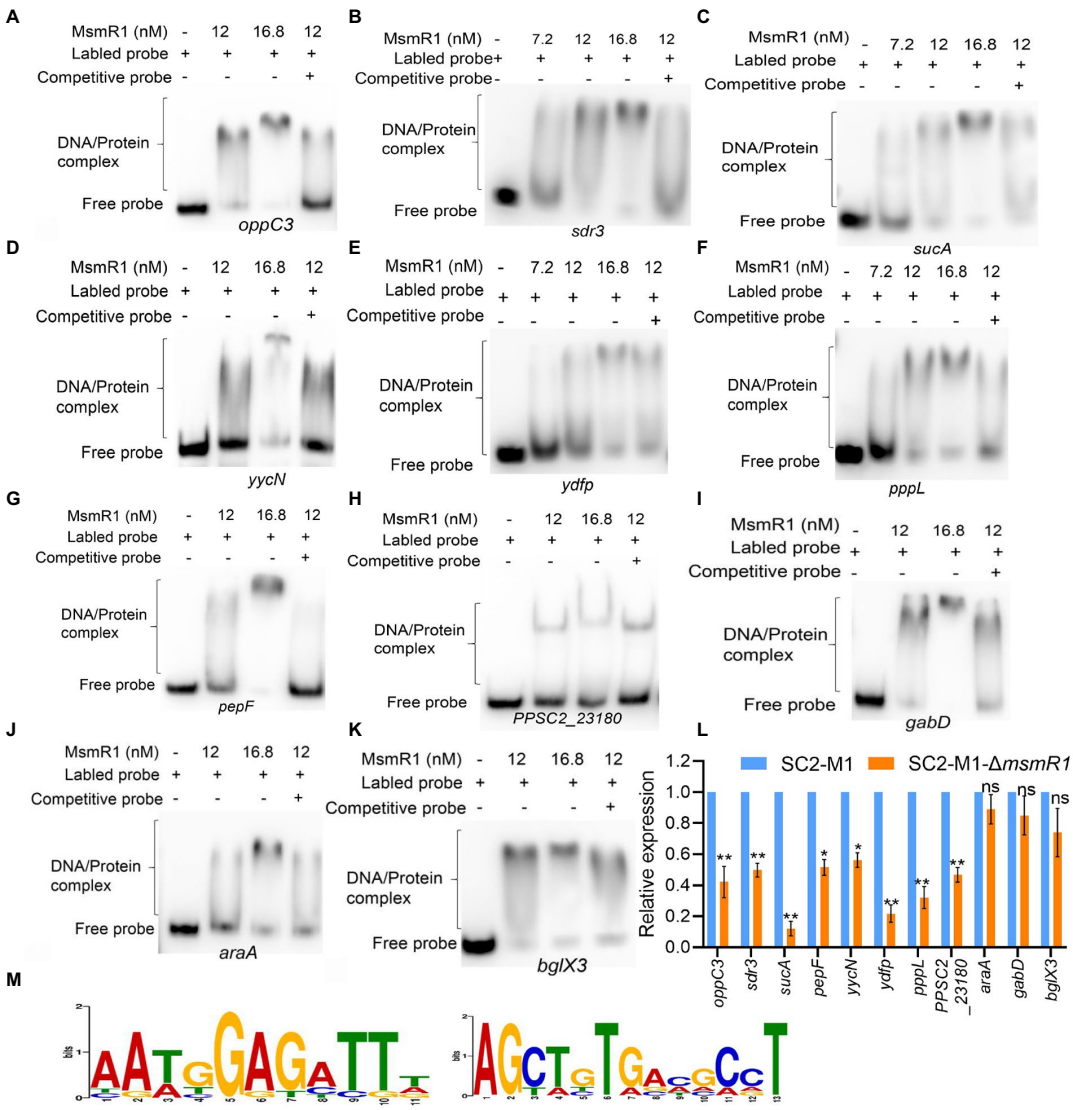
Identification of MsmR1 binding genes. EMSA results revealed that MsmR1 could bind directly to the upstream fragment of **(A)** *oppC3*, **(B)** *sdr3*, **(C)** *sucA*, **(D)** *yycN*, **(E)** *ydfp*, **(F)** *pppL*, **(G)** *pepF*, **(H)** *PPSC2_23180*, **(I)** *gabD*, **(J)** *araA* and **(K)** *bglX3*. **(L)** The relative expression of *oppC3*, *sdr3*, *sucA*, *pepF*, *yycN*, *PPSC2_23180*, *pppL*, *ydfp*, *gabD*, *araA* and *bglX3* determined by qRT-PCR. **(M)** MsmR1 binding motifs identified by MEME analysis. *p < 0.05, **p < 0.01 and ns, *p* > 0.05.

EMSA was performed to further validate the potential direct binding fragments with MsmR1. As shown in Figures 3A–K, MsmR1 could bind to the upstream fragments of *oppC3*, *sucA*, *sdr3*, *yycN*, *pepF*, *pppL*, *ydfp*, *PPSC2_23180*, *gabD*, *araA*, and *bglX3*. The qRT-PCR results revealed that compared with strain SC2-M1, the relative expression of genes *oppC3*, *sucA*, *sdr3*, *yycN*, *pepF*, *pppL*, *ydfp*, and *PPSC2_23180* were significantly decreased in *msmR1* mutant (*p* < 0.05, Figure 3L), which suggested that MsmR1 could positively regulate these genes. While no significant difference was observed in the expression of *gabD*, *araA*, and *bglX3* between SC2-M1 and SC2-M1-Δ*msmR1* (*p* < 0.05, Figure 3L). These results indicated that MsmR1 might not regulate the expression of *gabD*, *araA*, and *bglX3*, even though MsmR1 could bind to them. Additionally, we also determined two MsmR1-binding motif by MEME (Figure 3M).

### MsmR1 positively regulates polymyxin synthesis by directly binding to the *oppC3* and *sdr3* promoters

**Figure 4:**
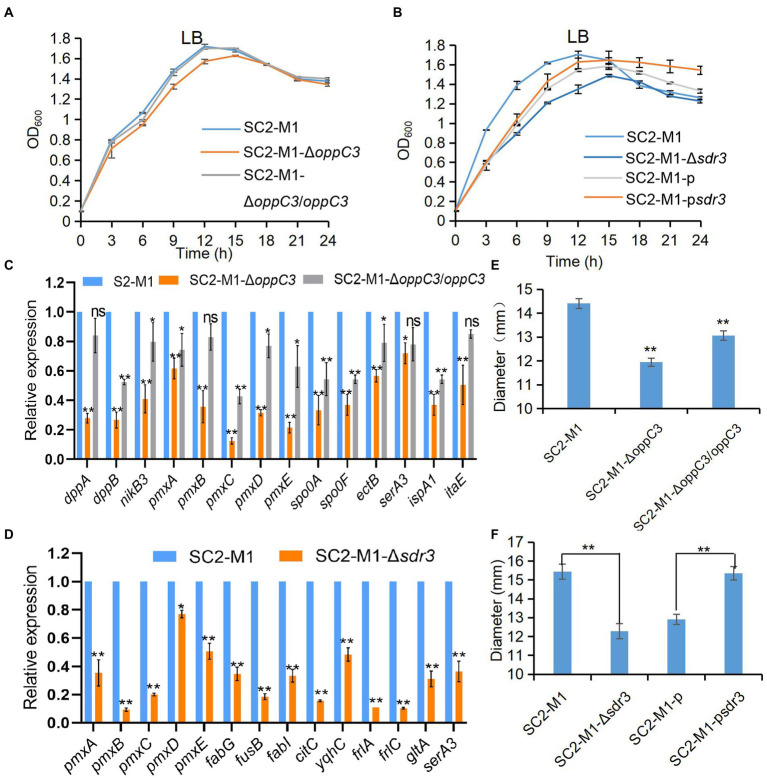
MsmR1 regulates polymyxin synthesis by directly binding to *oppC3* and *sdr3* promoters. **(A)** Growth curves of SC2-M1, SC2-M1-Δ*oppC3*, and SC2-M1-Δ*oppC3/oppC3* in LB medium at a wavelength of 600 nm. **(B)** Growth curves of SC2-M1, SC2-M1-Δ*sdr3*, SC2-M1-p, and SC2-M1-p*sdr3* in LB medium detected at a wavelength of 600 nm. **(C)** Relative expression of genes related to polymyxin synthesis gene cluster and amino acid transport and metabolism (*dppA*, *dppB*, *ectB*, *serA3*, *ispA1*, and *itaE*) in strain SC2-M1 and SC2-M1-Δ*oppC3* by qRT-PCR assay. **(D)** Gene expression related to polymyxin synthesis, amino acid metabolism (*gltA*, *serA3*), fatty acid metabolism (*fabG*, *fabI*, *fusB*) in strains SC2-M1 and SC2-M1-Δ*sdr3*. **(E)** Inhibition zone of the SC2-M1, SC2-M1-Δ*oppC3*, and SC2-M1-Δ*oppC3*/*oppC3* strains. **(F)** Inhibition zone of the SC2-M1, SC2-M1-Δ*sdr3*, SC2-M1-p, and SC2-M1-p*sdr3* strains. *p < 0.05, **p < 0.01 and ns, *p* > 0.05.

As described previously, carbohydrate and amino acid could affect the biosynthesis of polymyxin (Yu et al., 2018). The *oppC3* gene encodes a peptide ABC transporter together with the OppB protein forming a transmembrane channel that can transport and release peptides and nickel into cells (Ge et al., 2018; Slamti and Lereclus, 2019). The gene *sdr3* whose predicted product belongs to the short-chain dehydrogenase/reductase (SDR) family is important for carbohydrate, amino acid, lipid metabolism, and redox sensor mechanisms (Kavanagh et al., 2008). *oppC3* and *sdr3* affected the growth state of strain SC2-M1 in LB medium (Figures 4A,B). Therefore, we determined the expression levels of primary metabolism-related genes, such as those involved in carbohydrate, amino acid, and fatty acid metabolism (Supplementary Table S2), and all of these genes were downregulated in the mutant strains compared with SC2-M1 (Figures 4C,D). The growth state of SC2-M1-Δ*oppC3* and SC2-M1-Δ*sdr3* in basal medium with the sole carbon source supported their roles in carbohydrate metabolism (Supplementary Figures S4, S5). In the present study, polymyxin synthesis was significantly reduced in the knockout strains SC2-M1-Δ*oppC3* and SC2-M1-Δ*sdr3* compared with SC2-M1, and was recovered in the complement/overexpression strain (Figures 4E,F). Moreover, the qRT-PCR results confirmed that polymyxin synthetase genes were downregulated in the knockout strain compared with SC2-M1 (Figures 4C,D). The *oppC3* and *sdr3* genes indirectly influence polymyxin synthesis by reducing the rate of primary metabolism and nutrient uptake, and further reduce precursors and energy for polymyxin synthesis.

### MsmR1 positively regulates the TCA cycle by directly binding to the *sucA* promoter

**Figure 5:**
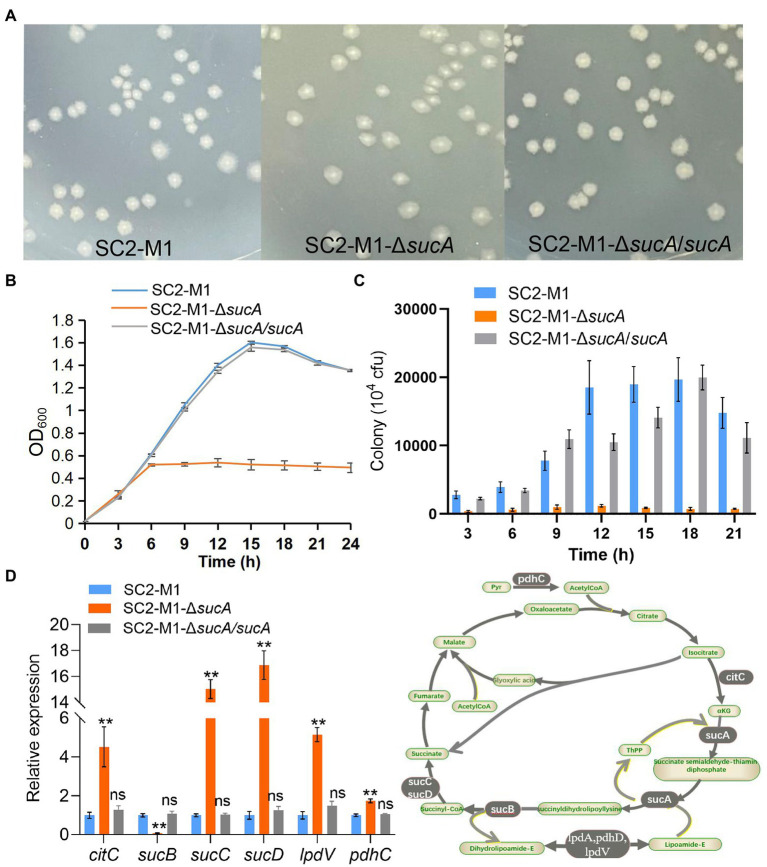
MsmR1 regulates carbohydrate metabolism by directly binding to the *sucA* promoter. **(A)** Colony morphology of the SC2-M1, SC2-M1-Δ*sucA*, and SC2-M1-Δ*sucA*/*sucA* strains in LB plates. **(B)** Growth curves of SC2-M1, SC2-M1-Δ*sucA*, and SC2-M1-Δ*sucA*/*sucA* in LB broth determined by OD_600_. **(C)** SC2-M1, SC2-M1-Δ*sucA*, and SC2-M1-Δ*sucA*/*sucA* colonies in LB broth determined by the live bacterial count method every 3 h. **(D)** Relative expression of genes related to the TCA cycle determined by qRT-PCR assay. **p < 0.01 and ns, *p* > 0.05.

The *sucA* gene encodes 2-oxoglutarate dehydrogenase (E1), part of the 2-oxoglutarate dehydrogenase complex (Gayán et al., 2019). In the TCA cycle, α-ketoglutarate is converted to succinate, which requires the catalytic action of the 2-oxoglutarate dehydrogenase complex (*sucAB*) and succinate dehydrogenase (*sucCD*; Walshaw et al., 1997). To assess the function of *sucA* in SC2, a knockout mutant strain SC2-M1-Δ*sucA* was generated. The colony morphology on LB plates appeared flat and transparent following *sucA* knockout, whereas the original morphology was recovered in the complemented strain SC2-M1-Δ*sucA*/*sucA* (Figure 5A).

The growth curve in LB medium was determined to evaluate the growth status, and showed that growth of the mutant strain SC2-M1-Δ*sucA* was markedly reduced compared to SC2-M1 and SC2-M1-Δ*sucA*/*sucA* (Figure 5B). Simultaneously, live bacterial counts were determined every 3 h; those of SC2-M1-Δ*sucA* were greatly reduced compared with those of SC2-M1 and SC2-M1-Δ*sucA*/*sucA*, which was consistent with the OD_600_ results (Figure 5C). Moreover, expression of the related gene *sucB* was markedly downregulated, while other genes involved in the TCA cycle, such as *sucC*, *sucD*, *citC*, *lpdV*, and *pdhC* (Supplementary Table S2) were markedly upregulated (Figure 5D). Due to disruption of the TCA cycle, bacteria may metabolize carbohydrate sources through the pentose phosphate pathway and glyoxylate cycle. Therefore, the energy decreased and the bacterial mass was further reduced.

During carbon source (xylose, arabinose, glucose, galactose, fructose, mannose, sucrose, maltose, trehalose, lactose and raffinose) utilization, the SC2-M1-Δ*sucA* strain presented a longer lag phase compared with the SC2-M1 strain. For the utilization of xylose, galactose, fructose, lactose, and raffinose, SC2-M1-Δ*sucA* presented a markedly lower OD_600_ value during the stationary phase compared with SC2-M1 (Supplementary Figure S6). Hence, *sucA* have essential roles in carbon source utilization.

### MsmR1 regulates motility, biofilm, and spore synthesis, as well as other processes

**Figure 6:**
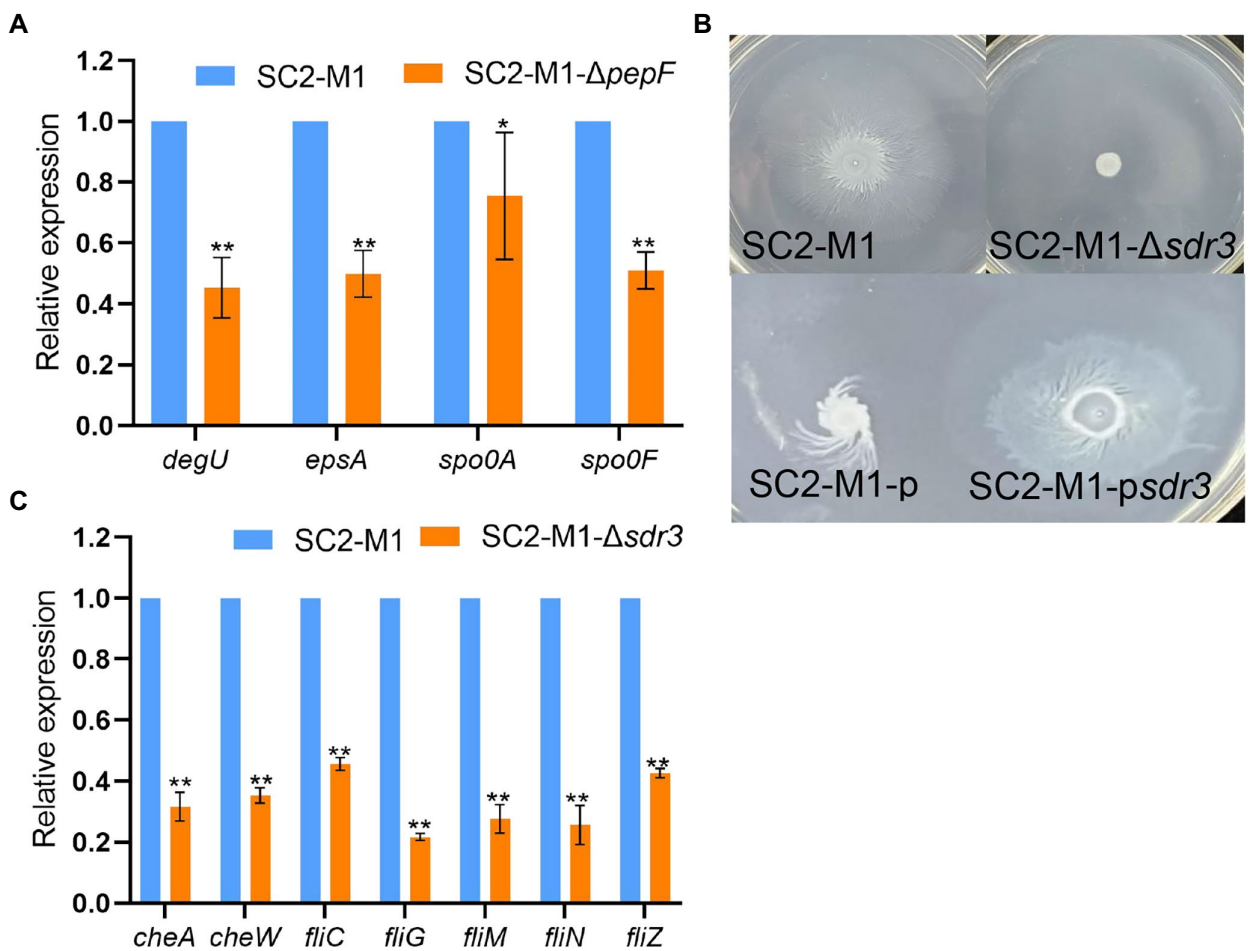
MsmR1 positively regulates motility, biofilm, and spore synthesis. **(A)** The expression of genes related to spore and biofilm formation in strain SC2-M1-Δ*pepF* and SC2-M1. **(B)** Motility of the SC2-M1, SC2-M1-Δ*sdr3*, SC2-M1-p, and SC2-M1-p*sdr3* strains in a specific plate. **(C)** The expression of genes related to chemotaxis and flagellum (*cheA*, *cheW*, *fliC*, *fliG*, *fliM*, *fliN*, *fliZ*) determined by qRT-PCR. *p < 0.05 and **p < 0.01.

The *pepF* gene encodes an oligoendopeptidase F. In *Bacillus subtilis*, the *pepF* homolog *phrA* can regulate spore formation (Kanamaru et al., 2002), and in *Aeromonas hydrophila*, *pepF* can regulate biofilm formation (Du et al., 2016). qRT-PCR results revealed that *pepF* regulated spore and biofilm formation. In other words, the relative expressions of genes related to biofilm and spore formation were downregulated (Figure 6A). The relative expressions of these genes were also downregulated in strain SC2-M1-Δ*msmR1* compared with SC2-M1 (Supplementary Figure S7). Overall, MsmR1 could positively regulate spore and biofilm formation.

Chemotaxis enables microbes to sense environmental signals and move to more favorable environments, which is important for bacterial colonization (Ud-Din and Roujeinikova, 2017). We found that motility was lost following *sdr3* deletion and was increased in the SC2-M1-p*sdr3* strain compared with that in the SC2-M1-p strain (Figure 6B). Moreover, the relative expression levels of genes encoding chemotaxis and flagellum (Supplementary Table S2) were downregulated in the SC2-M1-Δ*sdr3* strain compared with those in the SC2-M1 strain (Figure 6C). Taken together, these results indicate that MsmR1 may positively regulate cell motility by directly binding to the *sdr3* promoter.

The *yycN* gene encodes GNAT family acetyltransferase. The *ydfp* gene acts as a DoxX family protein, combined with SodA and SseA, forming a membrane-associated oxidoreductase complex (Nambi et al., 2015). The *pppL* gene encodes a PPM family protein phosphatase, which participates in environmental stresses (Shi et al., 1998). Hence, MsmR1 in *P. polymyxa* SC2 may also be involved in other biological processes.

## Discussion

**Figure 7:**
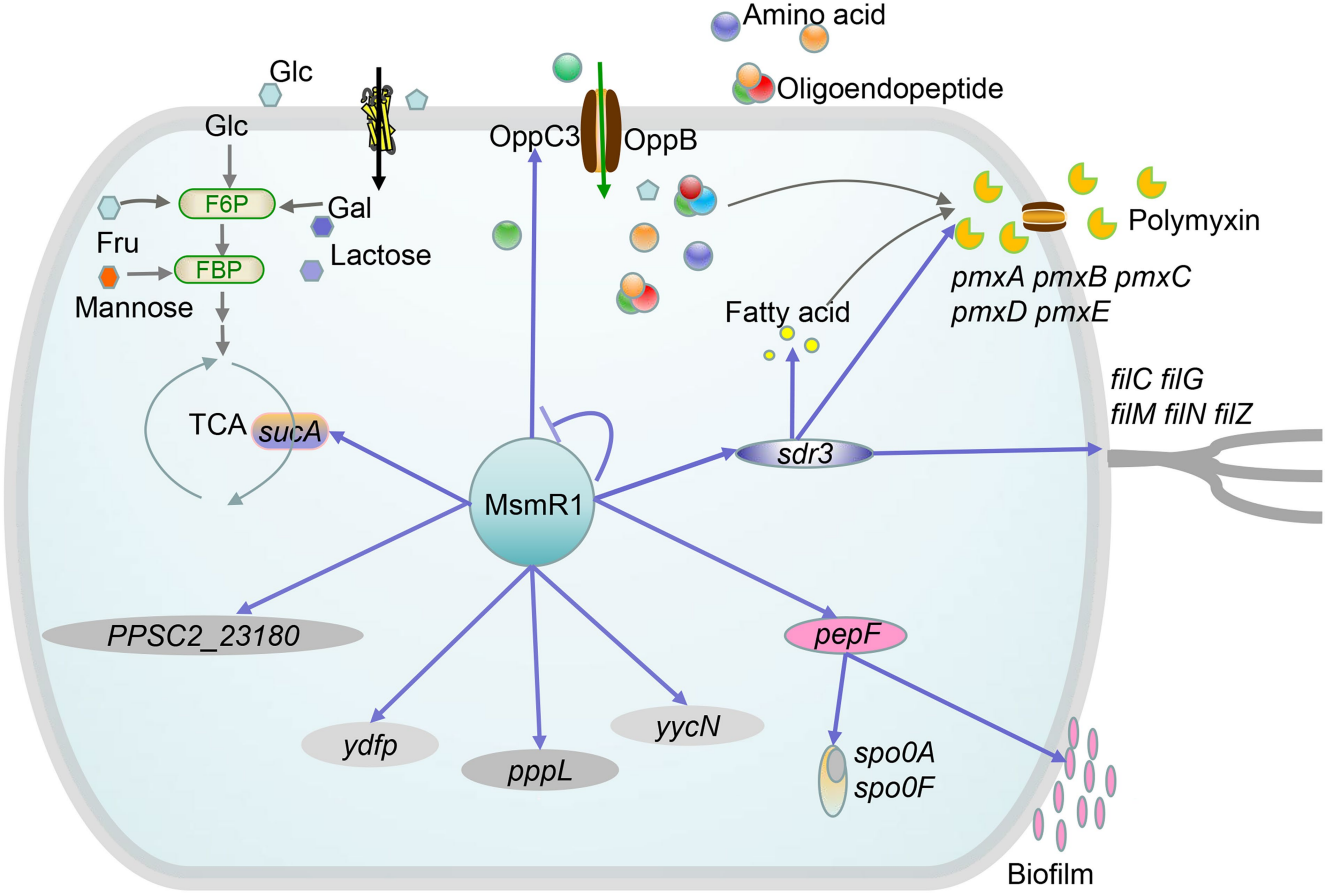
Proposed regulatory network of MsmR1 in *P. polymyxa* SC2. MsmR1 plays a regulatory role in the TCA cycle (*sucA*), amino acid metabolism, polymyxin synthesis (*oppC3*, *sdr3*), motility (*sdr3*), and biofilm and spore formation (*pepF*). Blue arrows: activation; bar-ended lines: repression; green arrows: metabolism process.

The purpose of this study was to elucidate the transcriptional regulatory mechanism of MsmR1 in *P. polymyxa* SC2. Sequence analysis revealed that MsmR1 comprises seven α-helices forming two HTH motifs at the C-terminal domain, which is the DNA binding domain, as well as a sensing or oligomerization domain of approximately 140 amino acids at the N-terminal domain. MsmR1 is a typical AraC/XylS family transcriptional regulator (Egan, 2002), which can act as activators, repressors, or both, and are involved in various biological processes ranging from metabolism to stress response and virulence (Gahlot et al., 2021). In this study, a ChIP-seq experiment combined with EMSA was performed, the regulatory network of the global regulator MsmR1 was proposed (Figure 7), and the role of MsmR1 in *P. polymyxa* was described.

MsmR1 directly and positively regulates the expression of *oppC3*, *sucA*, *sdr3*, *pepF*, *yycN*, *PPSC2_23180*, *pppL*, and *ydfp*. In particular, MsmR1 directly regulates *sucA* and influences the TCA cycle. OppC3, an ABC transporter, transports extracellular oligopeptides and oligosaccharides into cells, affecting primary metabolism and polymyxin synthesis. Meanwhile, as a member of the short-chain dehydrogenases/reductases (SDRs) family, Sdr3 can influence fatty acid, carbohydrate, amino acid metabolism, and motility, which in turn reduces energy and precursors for polymyxin synthesis. PepF affects spore and biofilm formation. Due to the fact that spontaneous mutant strain SC2-M1 lost the ability to form endospores and biofilm (Hou et al., 2016), we could not detect the physiological or biochemical characteristics of endospores and biofilm in the test strains. However, since the genes were not successfully knocked out, the functions of *PPSC2_23180*, *ydfp*, *pppL*, and *yycN* required further investigation. Nevertheless, the results of this study revealed the mechanism through which MsmR1 regulates polymyxin synthesis, carbohydrate metabolism, and other biological processes.

We obtained 649 peaks by ChIP-seq. KEGG enrichment analysis of these sequences revealed that the genes were involved in various functions, including cellular processes, environmental information processing, genetic information processing, and metabolism. Among these, the proportion of genes enriched in metabolism was the highest, reaching 81.25%, of which carbohydrate metabolism was the most enriched, followed by amino acid metabolism. These results support previous findings indicating that MsmR is primarily involved in polysaccharide metabolism (Russell et al., 1992). In addition, only 3.85% of the identified fragments were located in intergenic regions, whereas 96.15% were located in coding regions. This may be due to differences in the DNA-binding properties of MsmR1 *in vivo* and *in vitro*. Meanwhile, in *B. subtilis*, 643 binding sites for AbrB and 411 binding sites for Abh were identified, and approximately half of these binding sites were located in the gene coding region. Although half of all binding sites are located in intergenic regions, this does not affect transcription, even with strong binding intensities (Chumsakul et al., 2011). RutR is a regulator of pyrimidine catabolism in *E. coli*, and possesses 20 binding sites primarily bound to the gene coding regions with minimal effects on transcription levels (Shimada et al., 2008). Hence, MsmR1 may be a transcriptional regulator with binding site preferences that are similar to those of RutR.

Additionally, EMSA experiments revealed that MsmR1 could also bind its own promoter, however, this was not observed in the ChIP-seq results. In fact, most genes from the ChIP-seq results were not found to bind to MsmR1. There are several possible reasons for this. First, strains in the middle of the logarithmic growth phase were selected for ChIP-seq experiments; MsmR1 may interact weakly with these genes, and some binding sites may not have been detected (Galagan et al., 2013). Second, other cofactors may be required for MsmR1 to bind target genes. Transcription factors are typically coordinated by two or more transcription factors (Zhang et al., 2021). Third, adding a Flag tag to the C-terminus of MsmR1 with a *cat* resistance gene in the genome of SC2-M1 may interfere with the DNA binding ability and result in false-positive or -negative results. ChIP results reported for *Salmonella enterica* did not reveal previously described OmpR-binding targets but revealed that binding to some OmpR-regulated genes was affected by the C-terminal Flag tag (Perkins et al., 2013; Zhang et al., 2021). Finally, the EMSA conditions may influence the binding process. In the EMSA experiment, some retarded bands were relatively clear at low or high protein concentrations, while other DNA-MsmR1 binding processes presented clearer retarded bands only at high protein concentrations and smeared bands at low concentrations. This might be due to the different binding abilities of MsmR1 to DNA or the stability of the MsmR1-DNA complex *in vitro* during electrophoresis. As reported previously, a weak interaction occurs between the regulator VjbR and the *virB* (P*virB*) promoter; however, no retarded signal was observed in the EMSA experiment, indicating that VjbR and P*virB* cannot withstand the electrophoretic conditions of EMSA (Arocena et al., 2010). Therefore, in ChIP-seq, some DNA may have bound weakly to MsmR1 causing it to not be detected during EMSA electrophoresis.

MsmR is a regulator of polysaccharide metabolism, hence the growth curve of the *msmR1* knockout strain was determined. The results revealed no significant change in the LB medium or minimal medium with different carbon sources (Supplementary Figure S2E). However, its role in carbohydrate metabolism cannot be ruled out, as most of the identified target genes are related to carbon source metabolism. We hypothesized that this may be due to the functional redundancy of MsmR1 homologs in the SC2 strain. For genome sequence analysis, three genes were annotated as *msmR* (*msmR5*, *msmR7*, and *msmR9*), and 44 AraC family transcription factors were identified in the genome of *P. polymyxa* SC2, most of which were related to carbon source metabolism, such as arabinose metabolism and L-rhamnose metabolism (Supplementary Table S6). This possibility was verified using qRT-PCR, which revealed that *msmR5*, *msmR7*, and *msmR9* were all upregulated after *msmR1* knockout (Supplementary Figures S8A–C). Since *msmR5* was the most upregulated, we then assessed the impact of knocking out *msmR5*. The results showed no significant difference in SC2-M1, single mutant of *msmR5* (SC2-M1-Δ*msmR5*) and double mutant (SC2-M1-Δ*msmR1*/*msmR5*; Supplementary Figure S8D). In *Pseudomonas aeruginosa* PAO1161, PA3027 is an AraC-type transcriptional regulator with no effect on growth or carbon source utilization due to the functional redundancy of *glpD* homologs (Kotecka et al., 2021). Moreover, in *Ustilaginoidea virens*, due to the functional redundancy of other cutinase genes, cutinase activity was not affected after knockout of the cutinase G-box-binding protein UvCGBP1 (Chen et al., 2021). As a result of gene functional redundancy, growth status and carbon source utilization could not be considered the main markers of the *msmR1* knockout mutant.

AraC family transcription factors often form symmetric dimers and bind to palindromic DNA sequences (Kato et al., 2005; Sun et al., 2016). A 5-nt inverted repeat was identified by binding site analysis of the AraC family transcription factor SAV742, separated by 10 or 12 nt (GCCGA-n10/n12-TCGGC). Mutating the probe revealed that the 5-nt inverted repeat was essential for SAV742 binding (Sun et al., 2016). In *E. coli*, the TF RhaS could bind to the promoter region of *rhaI_1_*: ATCTTTCCCTGGTTGCC (Egan and Schleif, 1994). In *Pseudomonas putida*, Xyls could bind to two 15 bp direct repeats (TGCA-N6-GGNTA; Domínguez-Cuevas et al., 2008). In *Vibrio cholerae*, Toxt binds to a “toxbox” (yrTTTTwwTwAww). In the *tcpA*, *ctxAB*, and *tcpI-2* promoters, two “toxboxes” are direct repeats, while in the *acfD*/*acfA*, *tagA*, and *tcpI*-1 promoters, two “toxboxes” are inverted repeats (Weber and Klose, 2011). In *Streptomyces griseus*, the AdpA regulon consensus binding sequence is 5’-TGGCSNGWWY-3′, serving as inverted repeats at both ends, separated by 13–14 bp loops (Yamazaki et al., 2004). In contrast, *Streptococcus pyogenes* possesses two identical MsmR-binding motifs, ACTGTGACCATAA and GAATAGCTATTC, without palindromic sequences or inverted repeats (Nakata et al., 2005). In a study on the binding sequences (*emm*, *mrp*, *arp*, *enn*, and *scpA*) of Mga in *Streptococcus pyogenes*, a 45-bp binding site was identified by DNase I protection experiments, containing three conserved nucleotide regions and 29 nucleotides, separated by several unrelated nucleotides (Almengor and McIver, 2004). This consensus sequence failed to identify the location of the Mga-binding sites in the *mga*-promoter (McIver et al., 1999), suggesting that Mga can bind with different sequences (Almengor and McIver, 2004). In a study on the DNA-binding site of MsmR1 in the SC2 strain, two motifs were preliminary identified, while no palindromic sequences or inverted repeats were found. At the same time, no similar motifs were identified with those of other homologue MsmR1 proteins.

Oligopeptide permease (Opp) is primarily involved in importing short-chain peptides, such as dipeptide and tripeptide, from the extracellular environment (Solomon et al., 2003; Ge et al., 2018). The Opp system play an important role in the uptake of limiting nutrients like nickel, proline and betaine, especially under starvation and stress conditions (Alloing et al., 2006; Ge et al., 2018). Additionally, the Opp system plays a crucial role in various processes controlled by signal peptides, including sporulation, virulence, and binding (Slamti and Lereclus, 2019). In fact, OppC is required for peptide uptake but not for cell viability (Solomon et al., 2003). In this study, construction of an *oppC3-*knockout mutant revealed that *oppC3* also has an important role in amino acid metabolism. Simultaneously, the expression levels of genes related to nickel and sporulation were significantly downregulated following *oppC3* knockout. Polymyxin synthesis requires various amino acids as precursors. Owing to the downregulation of amino acid metabolism-related genes, precursor substances for polymyxin biosynthesis were reduced, further inhibiting the synthesis of polymyxin. In addition, *oppC3* may play an important role in oligosaccharide uptake by detecting the utilization of a sole carbon source. More specifically, *oppC3* plays a crucial role in the uptake of lactose and sucrose, but not maltose and trehalose. Meanwhile, with raffinose, a trisaccharide composed of glucose, galactose, and fructose, strains with *oppC3* knockout did not exhibit significant growth, suggesting that *oppC3* is critical for raffinose uptake. Hence, given that the role of *oppC* in the uptake of oligosaccharides has not yet been reported, the findings of this study report an important novel role of *oppC3* in the uptake of oligosaccharides.

During the interaction between *P. polymyxa* SC2 and pepper, the biosynthetic genes of polymyxin and fusaricidin were upregulated, which induced systemic resistance in pepper and enhanced its resistance to certain pathogens. Simultaneously, the upregulation of genes related to chemotaxis, motility, and biofilm formation is beneficial for the colonization of strains in the rhizosphere of plants (Liu et al., 2021). MsmR1, a global transcriptional regulator, regulates biological processes, including polymyxin synthesis, carbohydrate metabolism, biofilm formation, chemotaxis, and motility, which are beneficial for the interaction between *P. polymyxa* SC2 and pepper. The findings of this study provide a theoretical basis for polymyxin production and the application of strain *P. polymyxa* SC2.

## Conclusion

In the study, we investigated the transcriptional regulatory mechanism of MsmR1 in *P. polymyxa* SC2. MsmR1, a AraC/XylS family transcriptional regulator, is involved in polymyxin synthesis. In particular, MsmR1 was found to be highly associated with carbohydrate metabolism pathways. Indeed, MsmR1 affects polymyxin synthesis by directly regulating *oppC3* and *sdr3*, affecting basal metabolism and precursors for polymyxin synthesis, while also directly regulating *sucA* and influencing the TCA cycle. In addition, MsmR1 could influence spore and biofilm formation and other biological processes. These results provide a theoretical basis for the application of *P. polymyxa* SC2 in the biological control of various pathogenic bacteria in pepper.

## Data availability statement

The data presented in the study are deposited in the NCBI Sequence Read Archive (SRA) repository, accession number PRJNA885964.

## Author contributions

DZ performed the laboratory work, analyzed the data, and wrote the manuscript. HL, YC, and ST analyzed the data. CW, BD, and YD provided insights on the manuscript. YD and BD supported the study. All authors contributed to the article and approved the submitted version.

## Funding

This work was funded by the National Natural Science Foundation of China (31770115), Key Research and Development Program of Shandong Province (2021CXGC010804), and Tai-Shan Scholar Program from the Shandong Provincial Government.

## Conflict of interest

The authors declare that the research was conducted in the absence of any commercial or financial relationships that could be construed as a potential conflict of interest.

## Publisher’s note

All claims expressed in this article are solely those of the authors and do not necessarily represent those of their affiliated organizations, or those of the publisher, the editors and the reviewers. Any product that may be evaluated in this article, or claim that may be made by its manufacturer, is not guaranteed or endorsed by the publisher.
