# Supplementary material for: MsmR1, a global transcription factor, regulates polymyxin synthesis and carbohydrate metabolism in Paenibacillus polymyxa SC2
Source: Front Microbiol. 2022 Nov 22;13:1039806. doi: 10.3389/fmicb.2022.1039806 (PMC9722767; doi:10.3389/fmicb.2022.1039806)
Supplement: Supplementary file 2 [file Data_Sheet_2.docx]

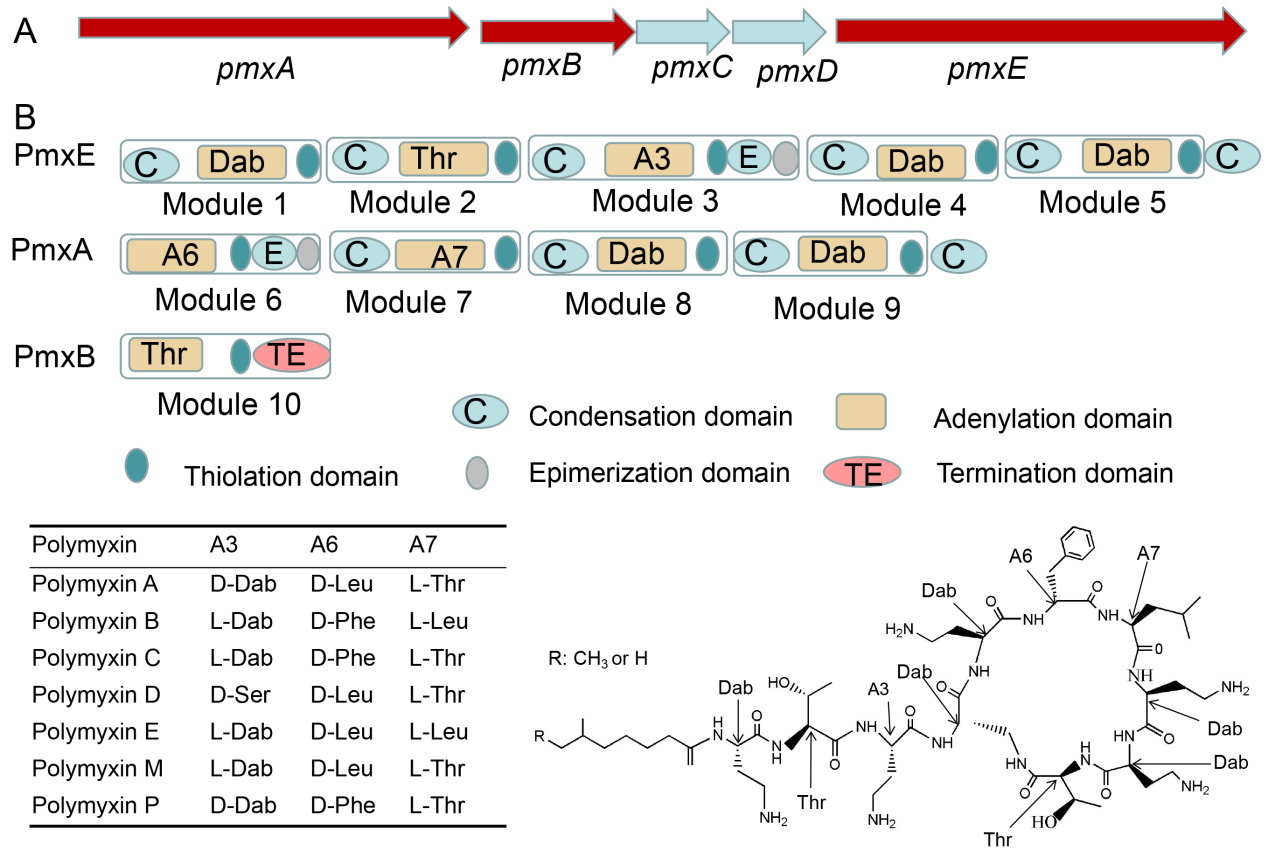


**Supplementary Figure S1 Schematic of the polymyxin biosynthesis from *P. polymyxa*.** (A) Gene cluster for polymyxin biosynthesis in *P. polymyxa* SC2. (B) Modules of polymyxin synthetase and basic structure of polymyxin in *P. polymyxa*.

B

C

A


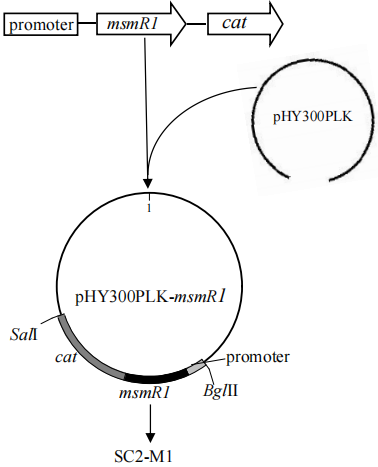

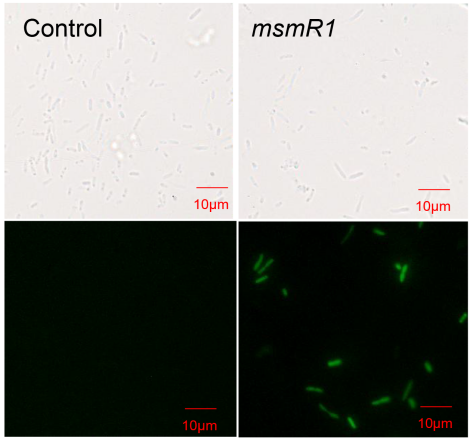

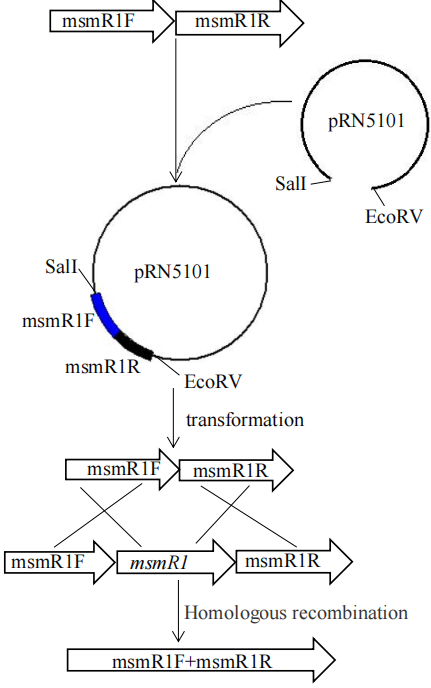


C


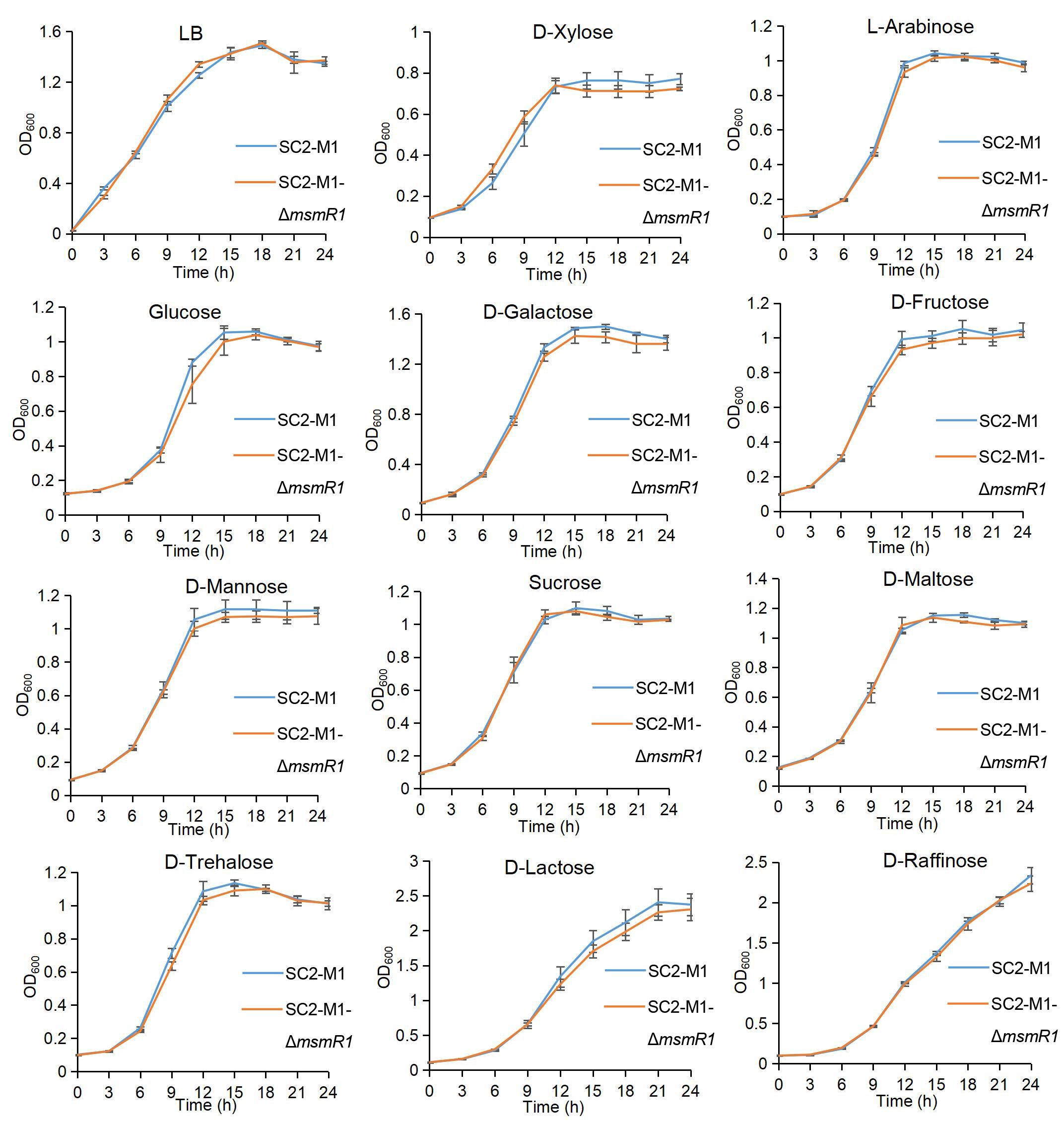


E


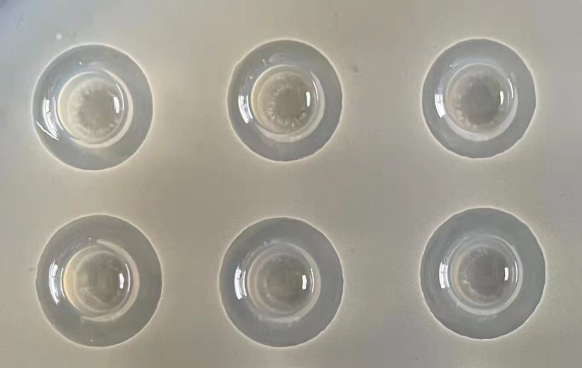

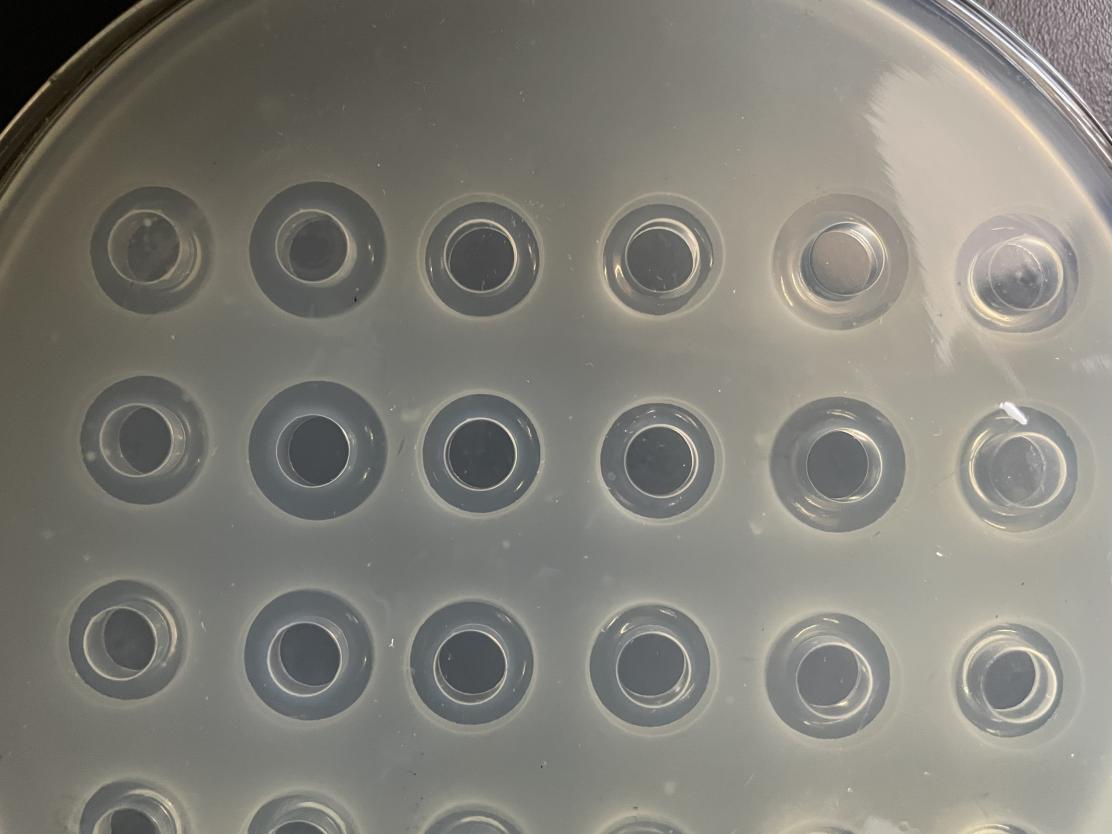


SC2-M1

SC2-M1-Δ*msmR1*

SC2-M1-p

SC2-M1-p*msmR1*

D

**Supplementary Figure S2 Knockout *msmR1* mutant characteristics.** (A) Fluorescence of P*msmR1* and control strains observed via a fluorescence microscope under blue light. The control (pHY300PLK-gfp) had no fluorescence, while the *msmR1* promoter (pHY300PLK-*msmR1*-gfp) showed fluorescence. (B) Strategy for *msmR1* gene knockout. (C) Strategy for overexpressing *msmR1*. (D) Inhibition zone of SC2-M1 and SC2-M1-Δ*msmR1*. (E) Growth curves of SC2-M1 and SC2-M1-Δ*msmR1* in Luria-Bertani (LB) broth and basal medium with sole carbon sources.

A

B

**Supplementary Figure S3** **Determination of the time point for ChIP-seq.** (A) Relative expression of *msmR1* at various time points using qRT-PCR. (B) Growth curve of SC2-M1 and SC2-M1-Flag.

*
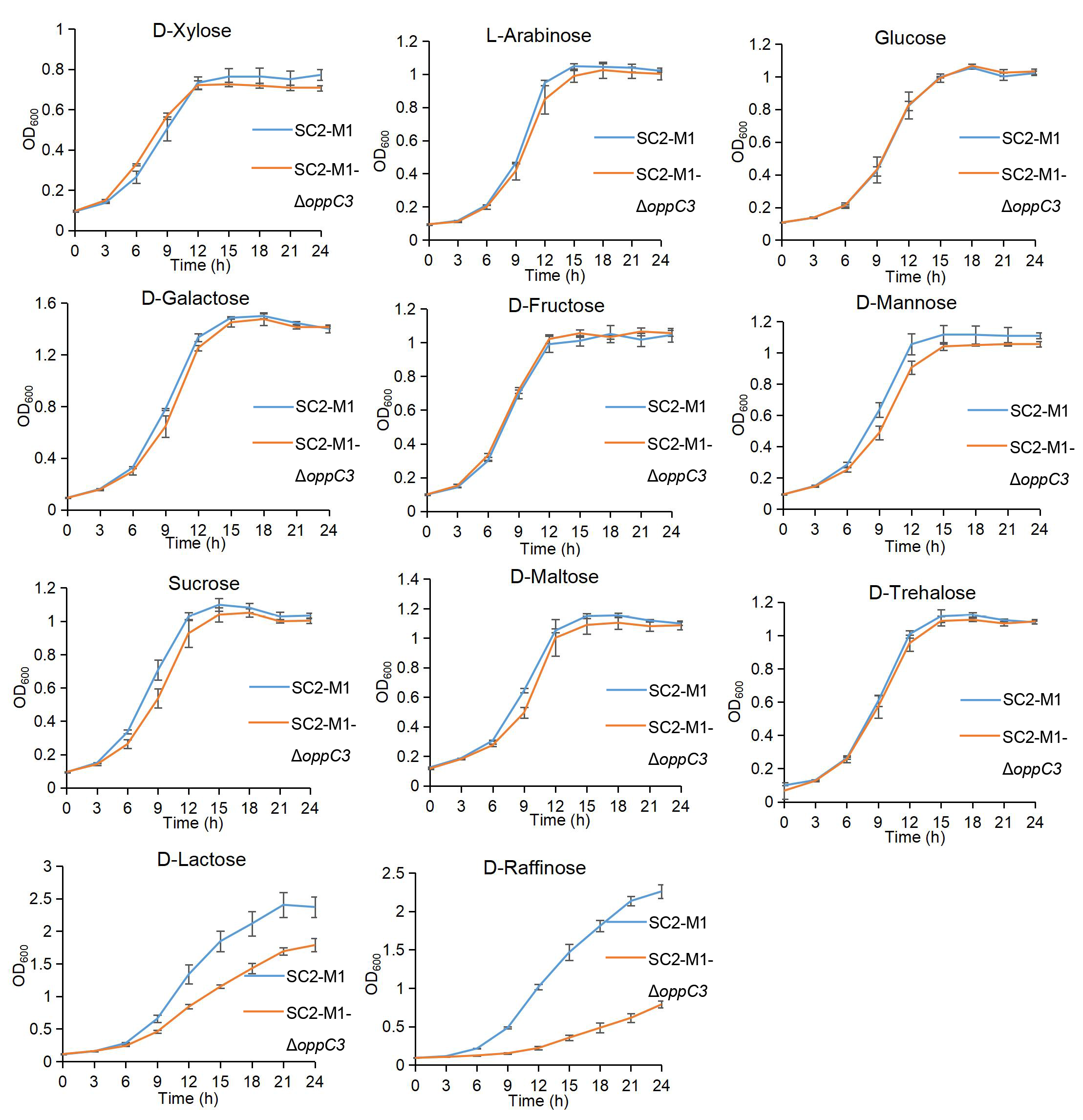
*

**Supplementary Figure S4 Growth state of SC2-M1-Δ*oppC3* in different medium.** Growth state of SC2-M1 and SC2-M1-Δ*oppC3* in basal medium with sole carbon sources determined by OD_600_ every 3 h.


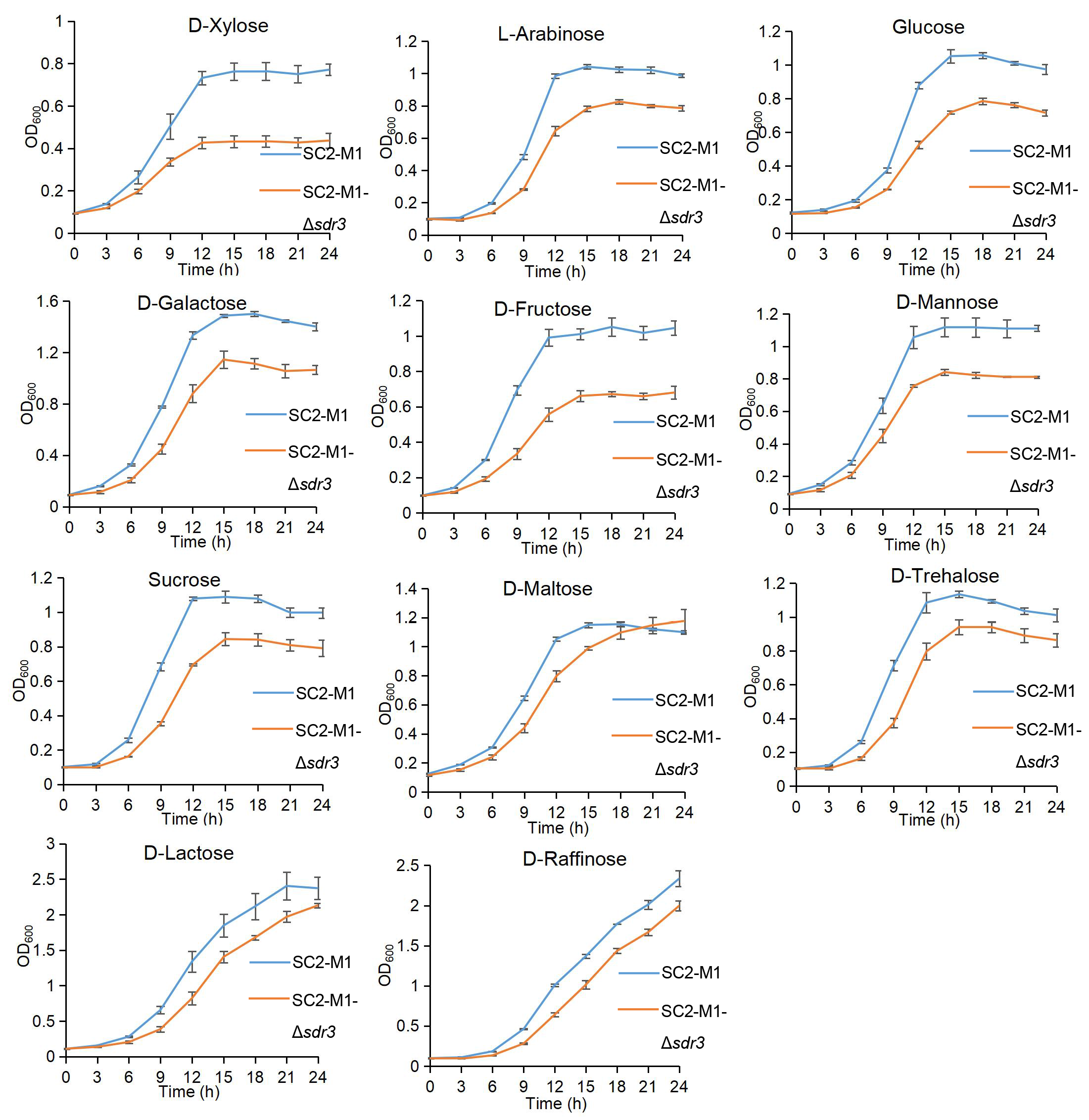


**Supplementary Figure S5** **Growth state of SC2-M1-Δ*sdr3* in basal medium with sole carbon sources.** Growth state of SC2-M1 and SC2-M1-Δ*sdr3* in basal medium with sole carbon sources determined by OD_600_ every 3 h.


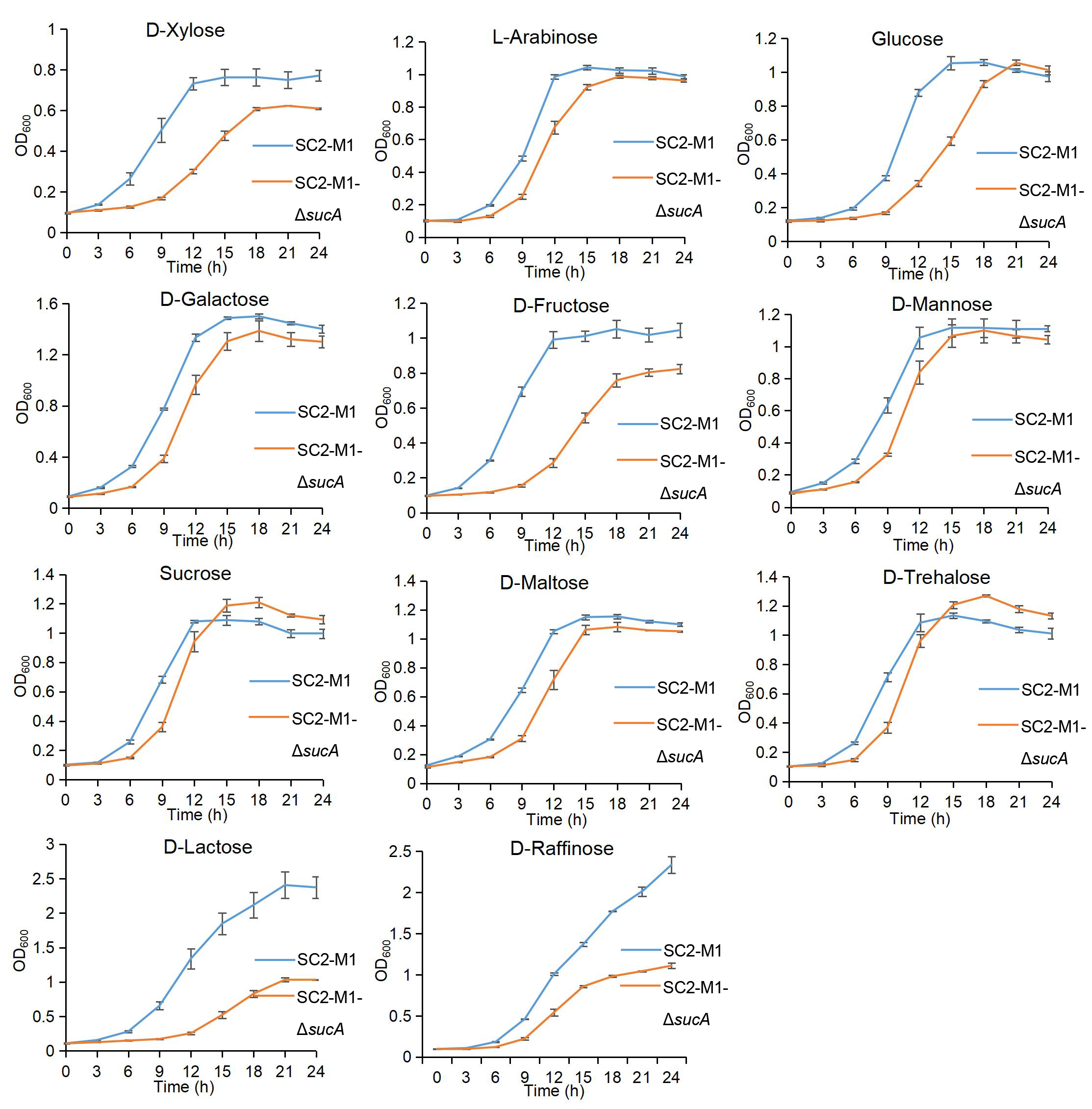


**Supplementary Figure S6** **The growth state of SC2-M1-Δ*sucA* in basal medium with sole carbon sources.** Growth state of SC2-M1 and SC2-M1-Δ*sucA* in basal medium with sole carbon sources were detected by OD_600_ every 3 h.


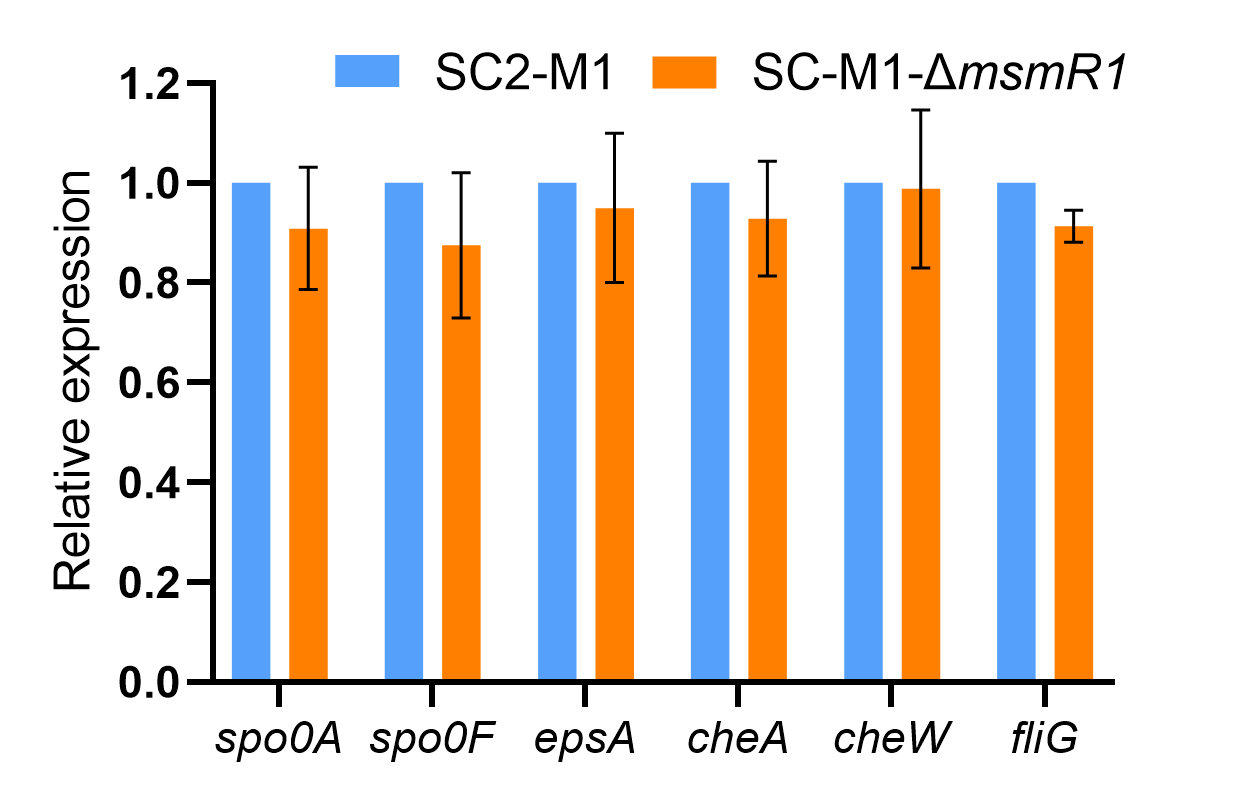


**Supplementary Figure S7** **MsmR1 regulates motility, biofilm, and spore synthesis.** Relative expression of spore and biofilm formation, chemotaxis related genes in strains SC2-M1-Δ*msmR1* and SC2-M1.


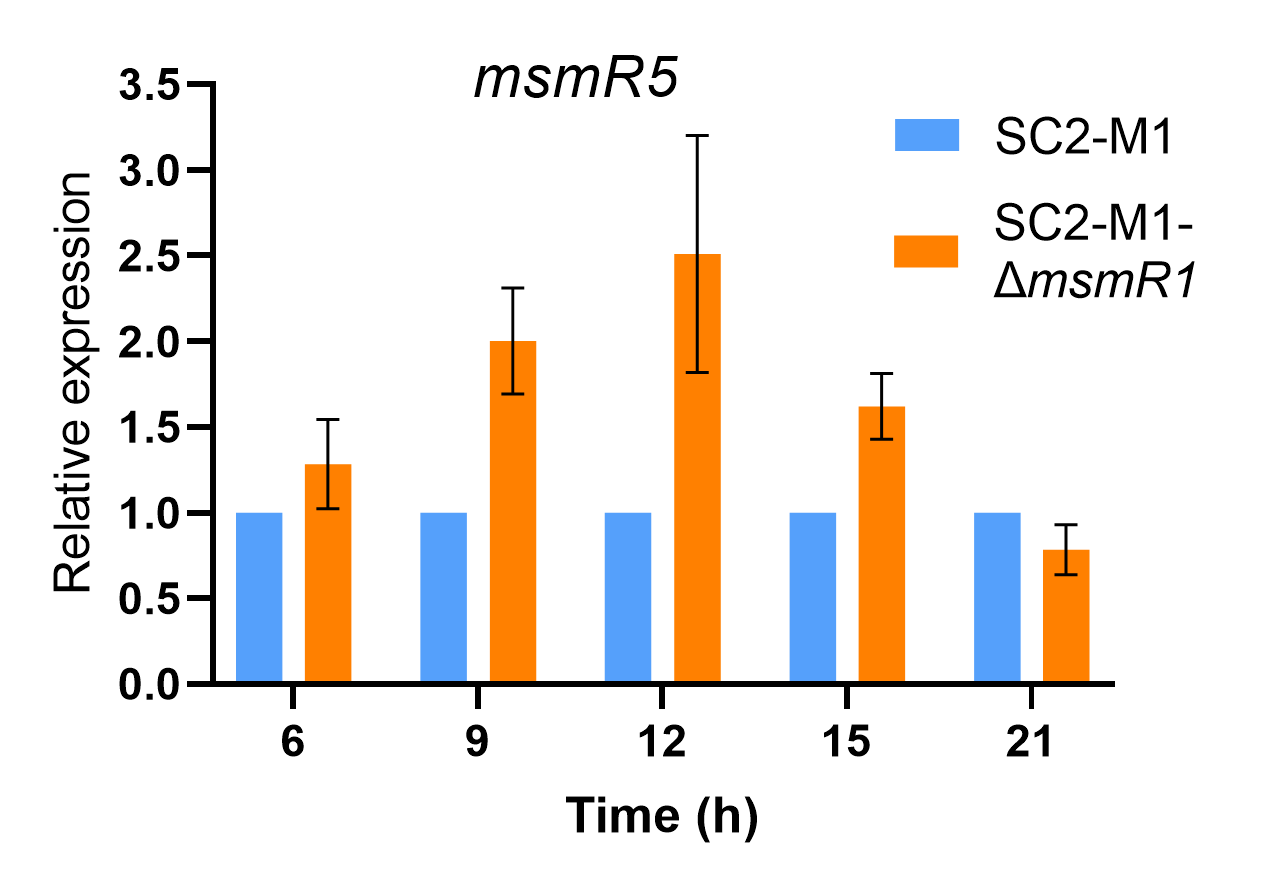

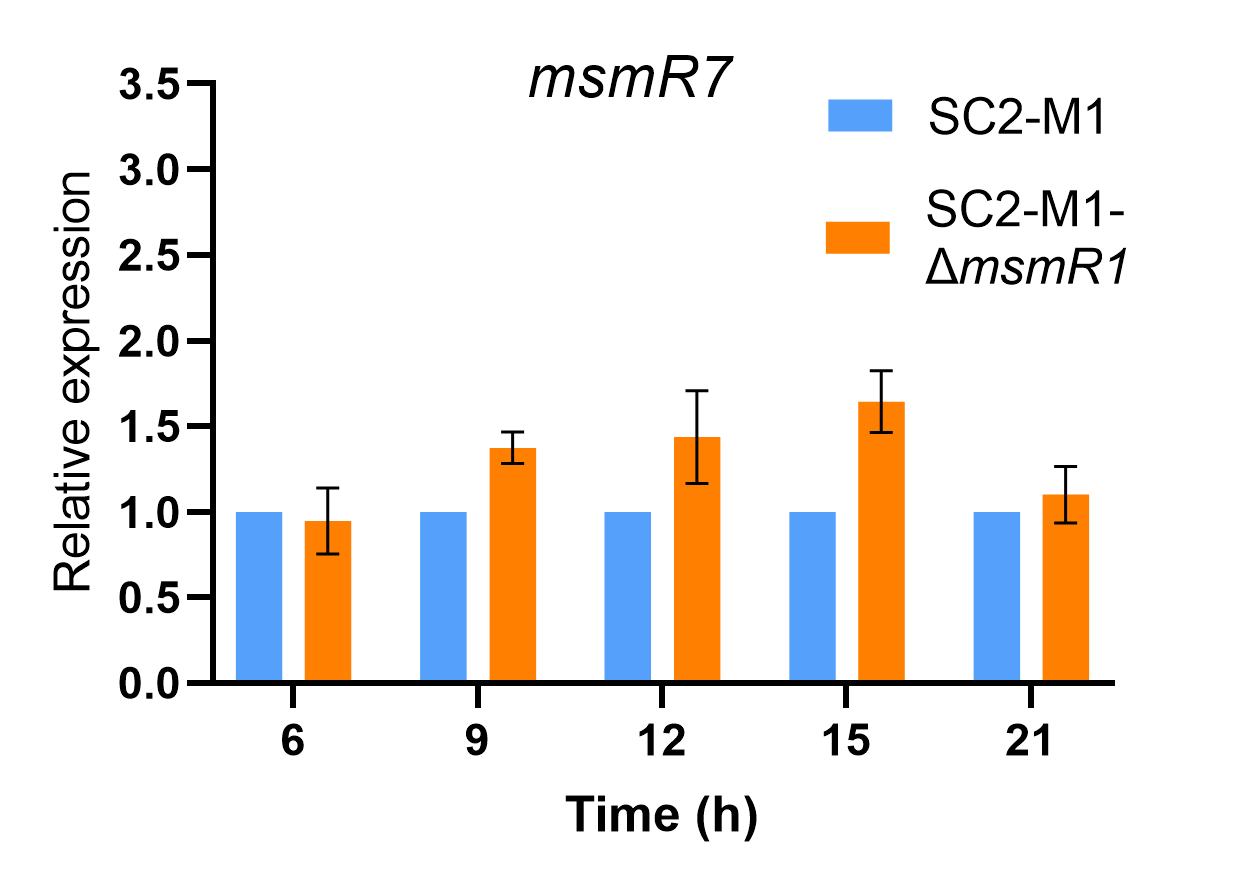


A

B

C


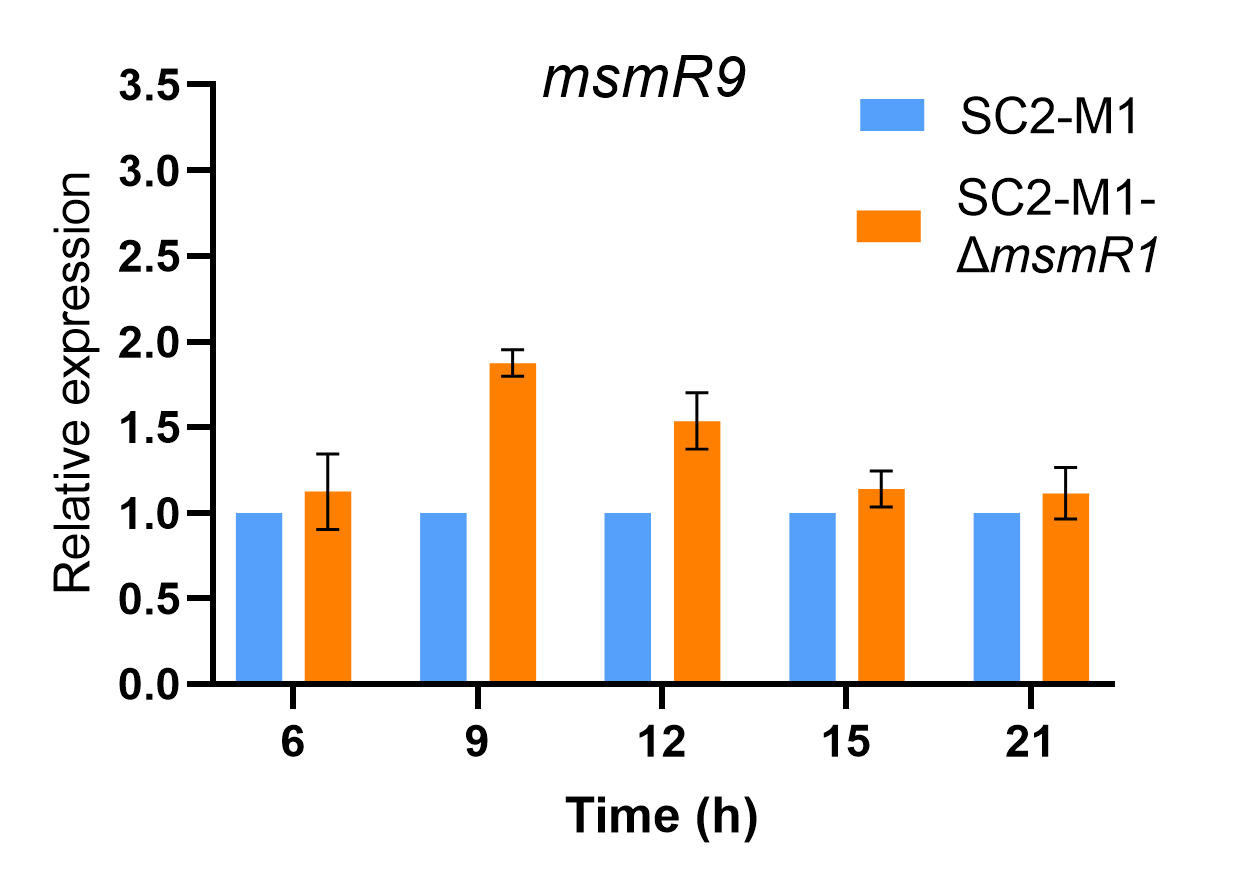

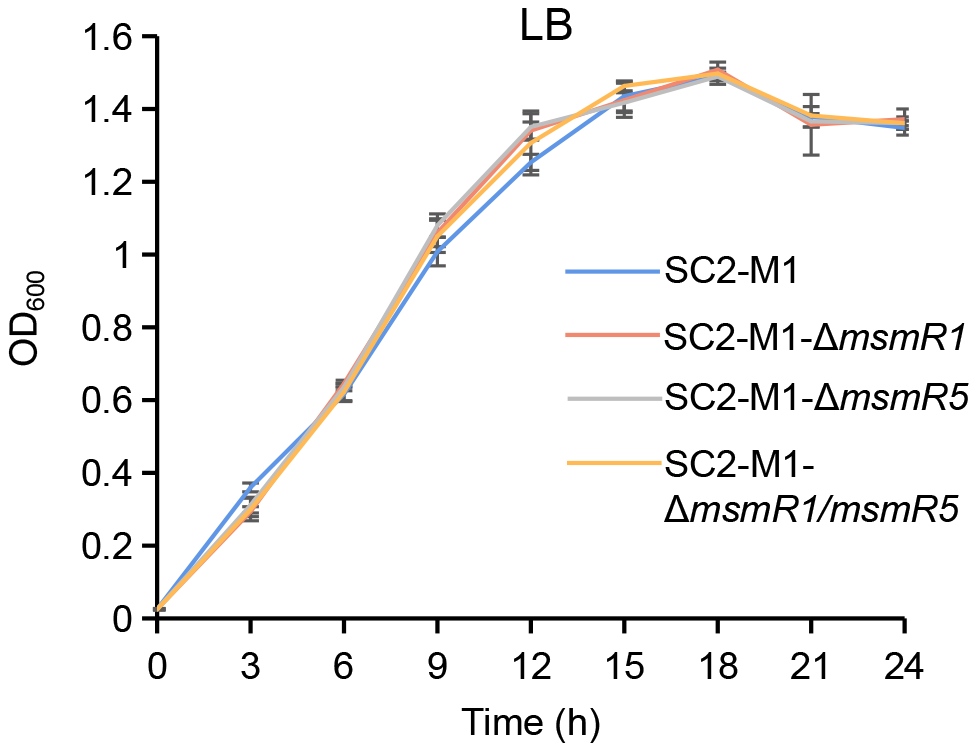


D

**Supplementary Figure S8** **Functional redundancy analysis of MsmR1 homologs in the SC2 genome.** (A–C) Relative expression of *msmR5*, *msmR7*, *msmR9* at various time points by qRT-PCR. (D) Growth state of strains SC2-M1, SC2-M1-Δ*msmR1*, SC2-M1-Δ*msmR5* and SC2-M1-Δ*msmR*1/*msmR*5 in LB medium determined via optical density at 600 nm every 3 h.
